# Measuring the Performance of Survival Models to Personalize Treatment Choices

**DOI:** 10.1002/sim.70050

**Published:** 2025-04-10

**Authors:** Orestis Efthimiou, Jeroen Hoogland, Thomas P. A. Debray, Valerie Aponte Ribero, Wilma Knol, Huiberdina L. Koek, Matthias Schwenkglenks, Séverine Henrard, Matthias Egger, Nicolas Rodondi, Ian R. White

**Affiliations:** ^1^ Institute of Primary Health Care (BIHAM) University of Bern Bern Switzerland; ^2^ Institute of Social and Preventive Medicine (ISPM) University of Bern Bern Switzerland; ^3^ Department of Epidemiology and Data Science Amsterdam University Medical Centers Amsterdam the Netherlands; ^4^ Smart Data Analysis and Statistics B.V. Utrecht the Netherlands; ^5^ Graduate School for Health Sciences University of Bern Bern Switzerland; ^6^ Department of Geriatric Medicine, University Medical Center Utrecht Utrecht University Utrecht the Netherlands; ^7^ Health Economics Facility, Department of Public Health University of Basel Basel Switzerland; ^8^ Institute of Pharmaceutical Medicine (ECPM) University of Basel Basel Switzerland; ^9^ Clinical Pharmacy and Pharmacoepidemiology Research Group Louvain Drug Research Institute (LDRI), UCLouvain Brussels Belgium; ^10^ Institute of Health and Society (IRSS) UCLouvain Brussels Belgium; ^11^ Population Health Sciences, Bristol Medical School University of Bristol Bristol UK; ^12^ Centre for Infectious Disease Epidemiology and Research, Faculty of Health Sciences University of Cape Town Cape Town South Africa; ^13^ MRC Clinical Trials Unit at UCL University College London London UK

## Abstract

Various statistical and machine learning algorithms can be used to predict treatment effects at the patient level using data from randomized clinical trials (RCTs). Such predictions can facilitate individualized treatment decisions. Recently, a range of methods and metrics were developed for assessing the accuracy of such predictions. Here, we extend these methods, focusing on the case of survival (time‐to‐event) outcomes. We start by providing alternative definitions of the participant‐level treatment benefit; subsequently, we summarize existing and propose new measures for assessing the performance of models estimating participant‐level treatment benefits. We explore metrics assessing discrimination and calibration for benefit and decision accuracy. These measures can be used to assess the performance of statistical as well as machine learning models and can be useful during model development (i.e., for model selection or for internal validation) or when testing a model in new settings (i.e., in an external validation). We illustrate methods using simulated data and real data from the OPERAM trial, an RCT in multimorbid older people, which randomized participants to either standard care or a pharmacotherapy optimization intervention. We provide R codes for implementing all models and measures.

## 1 Introduction

Clinical prediction models are widely used to inform and guide medical decision‐making and constitute useful tools for a wide range of diseases [1, 2]. Such models typically utilize a set of baseline participant characteristics to predict a future outcome, and several statistical and machine learning methods can be used for the task [3]. Assessing the predictive performance of such models is important for establishing their potential value and impact. Different methods and measures have been proposed for this assessment, covering model discrimination and calibration [4, 5]. In addition, decision‐analytic methods aim to assess the utility of prediction models, incorporating information on the possible clinical and economic consequences of decisions based on the models [6].

In recent years, there has been increased interest in predicting the effects of treatments at the individual level [7, 8], that is, predicting the relative benefits and harms for patients when treated with a new intervention as compared to a control treatment. Typically, data from randomized clinical trials (RCTs) are used to develop such models to reduce bias due to confounding. Many approaches can be used for this aim, utilizing methods from classical statistics, causal inference, machine learning, and hypothesis testing methods [9, 10, 11, 12]. Such models, if proven accurate, can inform personalized treatment recommendations and potentially lead to better patient outcomes. Assessing performance of models for personalized treatment choices, however, is difficult, because in RCTs usually each individual either receives treatment or control; this means that participant‐level benefit is unobservable (the “fundamental problem of causal inference” [13]). Moreover, a model that predicts outcomes in both treatment and control may perform well in predicting the absolute outcome in both treatment groups, but, contrary to intuition, this does not guarantee low error of treatment benefit predictions [14, 15]; for an illustrative (artificial) example see our previous work [16] and the appendix of this paper. Thus, when assessing the accuracy of predictions for treatment benefit, the use of methods that assess the accuracy of outcome predictions are not suitable; instead, such a performance assessment should focus instead on the predicted benefit.

This situation has motivated the development of methods specifically for assessing a model's capacity to predict treatment benefit. Rolling and Yang [15] described a cross‐validation method aimed at selecting models that minimize treatment effect estimation errors. Schuler et al. [17] summarized the general framework and compared some methods in simulations. Skrivankova and Heagerty [18] proposed a smooth non‐parametric evaluation method for assessing the performance of treatment rules. Van Klaveren et al. [19] introduced a concordance statistic for benefit, a generalization of the usual c‐index. Maas et al. [20] presented a range of metrics for models designed to predict treatment effect, extending definitions of E‐statistics, cross‐entropy, and Brier score. Kapelner et al. [21] developed software for automating the assessment of performance, based on k‐fold cross validation. Sadatsafavi et al. [22] proposed the concentration of benefit index, a metric that quantifies the capacity of baseline covariates to explain observed heterogeneity of treatment effects and find individuals who will benefit the most from treatment. Kuhlemeier et al. [23] described a generic procedure for validating predictions of individual treatment effects in new samples, with respect to continuous outcomes; their method was based on a matching procedure. Xu and Yadlowsky [24] introduced a method for measuring the calibration error for models predicting heterogeneous treatment effects. Yadlowsky et al. [25] also proposed a range of metrics (rank‐weighted average treatment effect, RATE metrics) for comparing and testing the quality of treatment prioritization rules. Further, Vickers et al. [26] proposed a method for assessing the clinical value of a model for treatment benefit, capturing also the disutility of treatment (e.g., due to adverse effects or costs).

We also recently presented a set of methods for assessing the performance of prediction models for estimating participant‐level treatment effects, focusing on the case of binary and continuous outcomes, which serve as the starting points of this paper [16, 27]. We proposed distinguishing two dimensions of model performance, that is, discrimination‐for‐benefit and calibration‐for‐benefit. We also introduced a new concept, decision accuracy, which combines the two dimensions and quantifies the population‐level outcomes after using a model for participant‐level benefit to guide decisions. In the current work, we extend a key subset of previously proposed methods to cover the case of survival (time‐to‐event) outcomes. We focus on the scenario when data from an RCT are used to develop a model for predicting participant‐level treatment effects on a survival outcome, and we want to assess the quality of these predictions. The methods are described for the case of internal validation, that is, following model development. However, they can be also readily applied during external validation, that is, when testing a pre‐developed model using new data. Some of these methods only apply to models that predict outcomes under both treatment and control; however, most of our methods can be used even for models that only predict the benefit of the new intervention.

We start by introducing notation and providing definitions in Section 2. We describe the synthetic and real data we use to illustrate the methods in Section 3. In Section 4, we propose methods for assessing the performance of survival models predicting participant‐level treatment effects, and in Section 5, we present results from the analyses of our examples.

## 2 Background and Definitions

### 2.1 Notation

We assume that we have a complete dataset (i.e., no missing baseline data) from a randomized trial that randomized $N_{p}$ participants to either treatment or control, with treatment denoted as $Z_{i}=1/0$ for each individual i. We also assume that we observed a vector of baseline covariates $x_{i}=\left(x_{i1},x_{i2}\ldots \right)$. Data are assumed to be right‐censored, that is, we either observed the time to a binary event of interest or the time of longest event‐free follow‐up; we denote this time as $T_{i}$. We denote the occurrence of an event as $C_{i}=1$, and use $C_{i}=0$ if no event occurred before or on $T_{i}$. We adopt the ICH E9 (R1) addendum (https://www.gmp‐navigator.com/files/guidemgr/E9‐R1_Step4_Guideline_2019_1203.pdf) on estimands and sensitivity analysis in clinical trials to address the presence of intercurrent events, focusing on the following: treatment policy, composite variable, and while‐on‐treatment. Estimating these is straightforward using randomized data as they do not require additional analysis methods and merely ignore (treatment policy) or incorporate intercurrent events in the outcome definition or in the censoring time. Without loss of generality, we assume the event to be harmful (e.g., death).

Next, for each individual we postulate the existence of two unobserved, underlying functions of time t, corresponding to the “true” survival probability under treatment or control; we denote them as $S_{i}^{1}\left(t\right)$ and $S_{i}^{0}\left(t\right)$. These probabilities are assumed to be conditional on a (possibly infinite) set of participant‐level covariates, of which only a subset was observed (i.e., $x_{i}$). Note that although we used the word “survival” throughout the paper, methods are applicable for any type of binary outcome, not necessarily death.

Finally, we assume that using this data we develop a multivariable model M that uses baseline covariates $x_{i}$ as input and predicts the survival probability function of individual patients under treatment and control. This model can be any statistical or machine learning model. We denote model predictions for individual i as ${\hat{S}}_{\mathrm{iM}}^{1}\left(t\right),{\hat{S}}_{\mathrm{iM}}^{0}\left(t\right)$ respectively. Alternatively, a model may predict benefit (see definitions below) directly, without estimating ${\hat{S}}_{\mathrm{iM}}^{1}\left(t\right),{\hat{S}}_{\mathrm{iM}}^{0}\left(t\right)$. Table 1 provides an overview of the notation used in this paper.

**TABLE 1:** Table of notation used in this paper.

Notation	Description
i	Participant indicator
$x_{i}=\left(x_{i1},x_{i2}\ldots \right)$	A set of participant‐level covariates for participant i
$T_{i}$	Total follow‐up time for participant i. This is either the time to the event of interest, or the time of censoring
$C_{i}$	Censoring indicator. This is either 0, if the participant was censored before the event happened, or 1
$Z_{i}$	Treatment indicator: 1 for treatment, 0 for control
$S_{i}^{1}\left(t\right)$ and $S_{i}^{0}\left(t\right)$	True survival probabilities under treatments 1 and 0 as a function of time t
$B_{i}^{\mathrm{SP}}\left(t\right)$	Benefit in survival probability $B_{i}^{\mathrm{SP}}\left(t\right)=S_{i}^{1}\left(t\right)-S_{i}^{0}\left(t\right)$
$B_{i}^{\mathrm{ST}}$	Benefit in survival time $B_{i}^{\mathrm{ST}}=\int_{0}^{\infty }\left(S_{i}^{1}\left(t\right)-S_{i}^{0}\left(t\right)\right)\mathrm{dt}$
$B_{i}^{\mathrm{RST}}\left(t_{*}\right)$	Benefit in restricted survival time $B_{i}^{\mathrm{RST}}\left(t_{*}\right)=\int_{0}^{t_{*}}\left(S_{i}^{1}\left(t\right)-S_{i}^{0}\left(t\right)\right)\mathrm{dt}$
$B_{i}^{\mathrm{PS}}\left(p\right)$	Benefit in percentile survival. $B_{i}^{\mathrm{PS}}\left(p\right)=t_{i}^{1}\left(p\right)-t_{i}^{0}\left(p\right)$, where $t_{i}^{1}\left(p\right)$ is the time at which $S_{i}^{1}\left(t_{i}^{1}\left(p\right)\;\right)=p$ where 0<p<1
$HR_{i}\left(t\right)$	Hazard function for each participant under each treatment as $HR_{i}\left(t\right)=h_{i}^{1}\left(t\right)/h_{i}^{0}\left(t\right)$, where $h_{i}^{1}\left(t\right)=-\frac{1}{S_{i}^{1}\left(t\right)}\;\frac{d\left(S_{i}^{1}\left(t\right)\right)}{\mathrm{dt}}$ and $h_{i}^{0}\left(t\right)=-\frac{1}{S_{i}^{0}\left(t\right)}\;\frac{d\left(S_{i}^{0}\left(t\right)\right)}{\mathrm{dt}}$
M	A model that uses participant covariates ($x_{i}$) to make predictions about survival probabilities under each treatment
${\hat{S}}_{\mathrm{iM}}^{1}\left(t\right)$, ${\hat{S}}_{\mathrm{iM}}^{0}\left(t\right)$	Predicted survival probabilities for participant i at time t under model M
${\mathrm{PB}}_{M}$, ${\mathrm{PB}}_{\mathrm{MvC}}$, ${\mathrm{PB}}_{\mathrm{MvT}}$	Population Benefit, a measure for decision accuracy. ${\mathrm{PB}}_{M}$ measures population‐level benefit of following model M to make decisions versus making decisions after doing the opposite of what the model suggests. ${\mathrm{PB}}_{\mathrm{MvC}}$ (resp. ${\mathrm{PB}}_{\mathrm{MvT}}$) measures benefit of following model versus treating no‐one (resp. everyone)
${\mathrm{PB}}_{M}^{\mathrm{SP}}\left(t_{*}\right)$, ${\mathrm{PB}}_{\mathrm{MvC}}^{\mathrm{SP}}\left(t_{*}\right)$, ${\mathrm{PB}}_{\mathrm{MvT}}^{\mathrm{SP}}\left(t_{*}\right)$	Population Benefit using survival probability at time $t_{*}$
${\hat{p}}_{\mathrm{iM}}^{\mathrm{cll}.1}\left(t\right)$, ${\hat{p}}_{\mathrm{iM}}^{\mathrm{cll}.0}\left(t\right)$	Complimentary log–log transformation on the predicted probability of an event until time t under model M ${\hat{p}}_{\mathrm{iM}}^{\mathrm{cll}.1}\left(t\right)=\log\left(-\log\left(1-{\hat{S}}_{\mathrm{iM}}^{1}\left(t\right)\right)\right)$, ${\hat{p}}_{\mathrm{iM}}^{\mathrm{cll}.0}\left(t\right)=\log\left(-\log\left(1-{\hat{S}}_{\mathrm{iM}}^{0}\left(t\right)\right)\right)$
${\hat{B}}_{\mathrm{iM}}^{\mathrm{cll}}\left(t\right)$	Predicted survival benefit under model M on the log–log scale ${\hat{B}}_{\mathrm{iM}}^{\mathrm{cll}}\left(t\right)={\hat{p}}_{\mathrm{iM}}^{\mathrm{cll}.1}\left(t\right)-{\hat{p}}_{\mathrm{iM}}^{\mathrm{cll}.0}\left(t\right)$
$B_{{\hat{B}}_{M}<0}^{\mathrm{obs}}$	Observed treatment benefit for the subgroup of participants for whom predicted benefit from model M was negative

### 2.2 Definitions of Treatment Benefit

True treatment benefit at the individual level can be defined in different ways. In general, we can define treatment benefit as $g\left(S_{i}^{1}\right)-g\left(S_{i}^{0}\right)$, where g is some suitable function of the survival probability function. Below we present several options for this choice.

*Benefit in survival probability*: We define true benefit in terms of survival probability, that is, the increase or reduction of the probability of an event at time $t_{*}$ when taking treatment instead of control, as $B_{i}^{\mathrm{SP}}\left(t_{*}\right)=S_{i}^{1}\left(t_{*}\right)-S_{i}^{0}\left(t_{*}\right)$.

*Benefit in expected survival time*: We define benefit in expected survival time for each participant as $B_{i}^{\mathrm{ST}}=\int_{0}^{\infty }\left(S_{i}^{1}\left(t\right)-S_{i}^{0}\left(t\right)\right)\mathrm{dt}$.

*Benefit in restricted survival time*: We define benefit in terms of expected restricted survival time up to time $t_{*}$ for each participant as $B_{i}^{\mathrm{RST}}\left(t_{*}\right)=\int_{0}^{t_{*}}\left(S_{i}^{1}\left(t\right)-S_{i}^{0}\left(t\right)\right)\mathrm{dt}$.

*Benefit in percentile survival*: If we focus on the *p*th percentile of survival, we can define benefit as the time at which true survival probability is *p*% in treatment, minus the time at which it is *p*% in control. For example, if we focus on p=50, if for a participant true survival probability is 50% at month 6 for treatment and month 4 for control, then the benefit in median survival is 2 months. More formally $B_{i}^{\mathrm{PS}}\left(p\right)=t_{i}^{1}\left(p\right)-t_{i}^{0}\left(p\right)$, where $t_{i}^{1}\left(p\right)$ is the time at which $S_{i}^{1}\left(t_{i}^{1}\left(p\right)\;\right)=p$, with 0<p<1.

*Benefit in hazard ratio*: We can calculate the hazard function for each participant under each treatment as $HR_{i}\left(t\right)=h_{i}^{1}\left(t\right)/h_{i}^{0}\left(t\right)$, where $h_{i}^{1}\left(t\right)=-\frac{d\left(\log\left(S_{i}^{1}\left(t\right)\right)\right)}{\mathrm{dt}}=-\frac{1}{S_{i}^{1}\left(t\right)}\;\frac{d\left(S_{i}^{1}\left(t\right)\right)}{\mathrm{dt}}$ and similarly $h_{i}^{0}\left(t\right)=-\frac{1}{S_{i}^{0}\left(t\right)}\;\frac{d\left(S_{i}^{0}\left(t\right)\right)}{\mathrm{dt}}$. Based on this definition, we can define benefit as a (time‐dependent) hazard ratio, $B_{i}^{\mathrm{HR}}\left(t\right)=\frac{h_{i}^{1}\left(t\right)}{h_{i}^{0}\left(t\right)}$.

Using model M, which predicts ${\hat{S}}_{\mathrm{iM}}^{1}\left(t\right),{\hat{S}}_{\mathrm{iM}}^{0}\left(t\right)$, we can in principle calculate treatment benefit following any of the definitions above. Alternatively, a model might directly estimate treatment benefit, bypassing the estimation of $S_{i}^{1}\left(t\right),S_{i}^{0}\left(t\right)$. In this paper we will explore methods for evaluating the performance of such predictions. This entails comparing model predictions with “observations,” that is, estimations of treatment benefit that are not dependent on the model. This procedure may be challenging, as some of the definitions of treatment benefit mentioned above may not be directly estimable without additional assumptions or extrapolations. For example, we cannot readily estimate mean survival time in a dataset where the last observation in time was censoring and not event. Likewise, we cannot estimate median survival benefit if in a dataset at the latest follow‐up 80% of the participants survived. This may constrain which of the proposed definitions of treatment benefit can be used in practice and are relevant to particular applied problems.

Finally, as a side note, the Cox model, which is arguably the most commonly used model for time‐to‐event analysis, directly estimates hazard ratios (HRs). However, although popular and easy to estimate, HRs may not be optimal for causal inference [28, 29]. Thus, in this paper we do not use them further.

### 2.3 Dimensions of Model Performance for Absolute Survival

Before tackling model performance for treatment benefit, we briefly review model performance for absolute survival and distinguish between two dimensions of model performance: discrimination and calibration.

*Discrimination* refers to the model's ability to accurately categorize patients into distinct risk groups. A model demonstrating strong discrimination will ensure that a patient with a longer predicted survival indeed survives longer than a patient with a shorter predicted survival. If we focus on a specific time point, we can measure discrimination like for binary outcomes, for example, using the area under the receiver operating characteristic curve (AUC). This corresponds to “fixed time point discrimination” [30]. In that case, the ordering of survival times before the specific time point is ignored. Censoring of follow‐up times, however, complicates this assessment. Uno et al.'s [31] inverse probability of censoring weights can be used to account for censoring. Discrimination across all time points (“time range discrimination”) [30] can be assessed using Harrell's c‐statistic [4]. Uno's c‐statistic, which uses censoring weights, can also be used [32]. Moreover, a time‐dependent discrimination index has been proposed, motivated by the practical problem of evaluating discrimination of non‐proportional hazards models [33].

*Calibration* relates to the average agreement between observations and predictions. For a model of good calibration, among participants with an estimated survival of 80% after 1 year, survival was indeed 80%. Calibration can be assessed for a fixed time point by comparing the average predicted survival from the model with the “observed” survival, that is, the one estimated while accounting for censorship. The latter can be obtained from a Kaplan–Meier curve (“calibration in‐the‐large” at fixed time). Further, Crowson et al. [34] proposed a model‐based framework for assessing calibration for proportional hazard models predicting survival and Austin et al. [35] described methods for constructing calibration curves and obtaining numerical measures.

### 2.4 Dimensions of Model Performance for Treatment Benefit

In our previous work [16], we adapted the definitions of calibration and discrimination and proposed the following definitions for measuring the performance of models for benefit:

*Discrimination for benefit* refers to the ability of a model to rank‐order participants with respect to their treatment benefit. For a perfectly discriminating model M, for two participants i, j for whom $B_{i}>B_{j}$, then ${\hat{B}}_{\mathrm{iM}}>{\hat{B}}_{\mathrm{jM}}$, where benefit is defined using any one of the definitions of Section 2.2.

*Calibration for benefit* refers to the average agreement between predicted and true treatment effects. For a model perfectly calibrated for benefit, among participants for which we predicted benefit X (using one of the definitions of Section 2.2), the true benefit is indeed X.

We also introduced the concept of *decision accuracy*. Decision accuracy refers to the ability of a model to identify participants who would benefit by at least $B_{\mathrm{Th}}$ from receiving treatment (Z=1) rather than control (Z=0). $B_{\mathrm{Th}}$ is a zero or non‐zero treatment benefit threshold, above which treating participants is worthwhile. For example, we may want to treat participants only if the benefit in survival at 2 years is more than 1%, due to adverse effects and costs of the intervention. In this case decision accuracy would refer to the ability of the model to identify participants who would benefit by at least 1% in terms of their true survival probability in 2 years, if they received treatment.

In Section 4, we will describe methods and measures for assessing discrimination for benefit, calibration for benefit, and decision accuracy for the case of survival models.

## 3 Data Used to Illustrate the Methods

### 3.1 Simulated Dataset

To illustrate our methods, we simulated an RCT of 2000 participants. For each participant i we generated four baseline covariates ($x_{1i}\ldots x_{4i}$), of which two were continuous ($x_{1},x_{2}$), one categorical ($x_{3}$), and one binary ($x_{4}$). Each participant was randomized to treatment or control with a 50% probability. We generated participant‐specific event rates as fixed across time points using a linear function of the covariates, the treatment indicator, and all treatment‐covariate interactions. We generated the time of death for each patient by drawing from an exponential distribution given the participant‐specific event rate. Finally, we generated censoring time by drawing from a uniform distribution. The median follow‐up time of the simulated participants was 1.3 years, and 1021 events (51%) were recorded. For 1421 participants (71%), true event rates were higher for control rather than treatment, that is, the average expected treatment benefit was positive. More details are shown in Appendix A.1.

Using this dataset, we estimated a Weibull survival model with linear terms for all covariates and interactions between all covariates and treatment. The specified model's family contains the data generating model; this was intentional, to ensure that the methods for assessing model performance work as intended (i.e., performance assessment was not affected by model misspecification). This model was then used to predict individualized treatment effects.

### 3.2 The OPERAM Trial: Optimizing Therapy to Prevent Avoidable Hospital Admissions in Multimorbid Older Adults

The *Optimizing thERapy to prevent Avoidable hospital admissions in Multimorbid older people* (OPERAM) study was a multicenter, cluster randomized, controlled trial of 2008 hospitalized patients ≥ 70 years of age with multimorbidity (≥ 3 chronic medical conditions) and polypharmacy (use of ≥ 5 chronic medications). Patients were randomly assigned to either standard care or a structured pharmacotherapy optimization intervention to detect potentially inappropriate prescribing. This intervention was deployed by a doctor and a pharmacist jointly, using a clinical decision software system. Results of this trial have been published previously [36]. The endpoint we use in this paper is all‐cause mortality. For this outcome, the original analysis found only a weak signal for the efficacy of the intervention, HR = 0.90 [0.71; 1.13]. In this paper, we also use information on the following patient‐level covariates measured at baseline, including cardiovascular risk factors and cardiovascular treatments: age, gender, binary smoking status, BMI, systolic blood pressure, diabetes status, treatment of hypertension, number of medications, history of heart failure, statin use, diuretic use, and cardiovascular disease. These covariates were preselected by experts, as potential effect modifiers. We excluded 232 (12%) patients with missing baseline data, as our purpose was to illustrate new methods. In real applications, we would include such patients in the analysis, for example, after imputing missing values. We included 1776 patients: 860 in the treatment (161 deaths) and 916 in the control group (179 deaths).

## 4 Methods

### 4.1 Using Out‐of‐Sample Predictions for Internal Validation

This section is only relevant for the case of internal validation, that is, when we want to use the same data to both develop the model and assess its performance. In what follows, we assume that we have a single source of data, that is, from a single RCT. In this situation, if the same data are used to develop and test a prediction model, the resulting performance measures can be misleading due to possible overfitting and subsequent optimism [1]. This assessment will lead to the apparent performance being better than the actual performance, that is, the performance of the model when applied to new data from the same setting. One way to reduce such optimism is to split data into two parts, a development and a testing dataset. This method, however, requires abundant data [37]. Alternative methods for obtaining unbiased performance measures include bootstrapping and k‐fold cross‐validation. In a previous work [16] we advocated the use of k‐fold cross‐validation. The reason is that with k‐fold cross‐validation, treatment benefit for a participant is estimated without using post‐randomization data from this participant, that is, the model used to predict treatment benefit for a specific patient is not allowed to “see” this patient's outcome. Thus, it can be treated as a baseline variable, for example, to split participants into groups while respecting randomization. This allows us to then estimate treatment effects in these groups without worrying about confounding. It also ensures that for each individual, we have exactly one out‐of‐sample prediction. Alternatively, we can use bootstrapping and use out‐of‐bag predictions. In what follows, we assume that we assess model performance using 10‐fold cross‐validation. Note that this is only for assessing performance; the final model is estimated using the whole dataset.

Our methods and measures described below can also be directly used for the case of external validation without the need to use sample splitting or resampling techniques.

### 4.2 Discrimination for Benefit

The C‐for‐benefit metric introduced by van Klaveren et al. [19] provides a means of assessing the discriminative ability of models aiming to identify the potential benefits of treatment at the individual level. It differs from the traditional C‐statistic, which focuses primarily on predicting the absolute risk of an outcome. Like the traditional C‐statistic, however, it ranges from 0 to 1, where a value of 0.5 indicates a completely uninformative model and 1 indicates a perfect model.

To calculate C‐for‐benefit, we create pairs of “similar” participants, where one participant in each pair received treatment, the other control. We then measure the observed benefit for each pair as +1, −1, or 0 by comparing survival times. Benefit equals +1 (−1) if the shorter observed time‐to‐event was for the participant in the control (treatment) arm. In simpler terms, if the participant in the control arm survived for a shorter time than the treated participant, the observed benefit equals +1. If the outcome at the shorter follow‐up time of the pair is censored, the benefit is set to 0. As such, C‐for‐benefit measures the probability that “from two randomly chosen matched pairs with unequal observed benefit, the pair with greater observed benefit also has a higher predicted benefit.” Van Klaveren et al. [19] proposed two methods for creating pairs of similar participants, using observed covariates or the model's predicted benefit, while Maas et al. [20] proposed matching based on Mahalanobis matching. Several other variations and extensions of the C‐for‐benefit have been proposed, including different definitions of the predicted benefit within a pair [20], a model‐based approach that avoids the need for matching [38], and resampling methods in case of unbalanced sample sizes of treatment groups [16]. A comparison of different estimators and definitions of underlying estimands is available elsewhere [38].

Depending on what approach is used, the measure can be applied to either models predicting outcomes for both treatment and control or models that predict treatment benefit without predictions for each treatment group. In this paper, we use the C‐for‐benefit as originally proposed by van Klaveren et al. [19], since it readily extends to time‐to‐event data and can be used even by models that only predict treatment benefit. The flexibility is an advantage of C‐for‐benefit, but the metric also has several problems, such as the sensitivity to the matching procedure [27] and the fact that it is not a proper scoring rule, that is, the expectation of the metric is not maximized when the correct model is used [39]. Moreover, it shares some of the limitations of the C‐index for regular survival models (which predicts outcomes, not benefit), outlined by Hartman et al. [38]. More details are provided in the Discussion.

### 4.3 Calibration for Benefit

A simple method to assess calibration for benefit is to visually compare the difference in the average model predictions between each treatment group with the “observed” ones, that is, the differences between group‐specific Kaplan–Meier curves. For quantitative measures, we can define estimands of interest using the true (unobserved) benefit $B_{i}$, where $B_{i}$ can correspond to any of the definitions for benefit described in Section 2.2. First, we define the calibration‐for‐benefit intercept ($a_{0}$) and calibration‐for‐benefit slope ($a_{1}$) of the linear regression $B_{i}˜{\hat{B}}_{\mathrm{iM}}$. We can also consider the overall difference between true and estimated benefit, that is, mean bias $\mathrm{Bia}s_{M}=E\left(B_{i}-{\hat{B}}_{\mathrm{iM}}\right)$, the mean squared error $\mathrm{MS}E_{M}=E\left({\left(B_{i}-{\hat{B}}_{\mathrm{iM}}\right)}^{2}\right)$, and the root mean squared error, $\mathrm{RMS}E_{M}=\sqrt{E\left({\left(B_{i}-{\hat{B}}_{\mathrm{iM}}\right)}^{2}\right)}$. The latter combine aspects of calibration and discrimination. When choosing among $\mathrm{MS}E_{M}$ and $\mathrm{RMS}E_{M}$, the latter has the advantage of being in the same scale as the benefit.

For estimation, we split participants into groups according to their predicted benefit. Then we estimate the observed benefit and compare it with the benefit predicted for the group. We can visualize results in a scatter plot and fit a linear regression to estimate $a_{0}$ and $a_{1}$. We can also use the predicted‐observed pairs to estimate $\mathrm{MS}E_{M}$ and ${\text{RMSE}}_{M}$. In essence, given that treatment benefit is unobservable at the participant level, we use group‐level statistics to assess the performance at the individual participant level. ${\text{Bias}}_{M}$ can be estimated directly by comparing the observed benefit with the predicted one across all participants in the dataset. These approaches can be used even for models that predict treatment benefit only, without predicting outcomes for each treatment assignment.

Readers here should note that our definition of $\mathrm{MS}E_{M}$ (i.e., mean squared error between true and predicted benefit) is conceptually similar to the Brier score for models predicting binary outcomes (not benefit): Brier score is the mean squared difference between predicted probabilities and observed binary outcomes. In our setting, however, individual‐level treatment effects are unobservable, and it is challenging to estimate this quantity. Two possible solutions are (i) to estimate observed treatment effect in groups of individuals, some of which received treatment and some control, or (ii) to approximate the individual‐level treatment effect after matching individuals one‐to‐one, that is, using procedures that create similar treatment‐control pairs of individuals. In this paper we follow the first approach. Maas et al. [20] followed the second approach and defined the “*Brier‐for‐benefit*” as the mean squared difference between predicted and observed benefit in pairs of “similar” patients, one of which took treatment and one of which took control. Their definition of Brier‐for‐benefit was not linked to an estimand, since it was made in conjunction with the estimation procedure (i.e., matching patients). However, it can be considered as an alternative estimate of $\mathrm{MS}E_{M}$, in addition to the estimate via clustering that we described above.

An alternative way to assess calibration is a model‐based approach, which avoids splitting the data into groups. In what follows we describe an approach that resembles the method proposed by Austin et al. [35], for the case when interest lies in treatment benefit. This method requires that the model provides estimates of survival under both treatment and control, that is, it cannot be used by models that only predict benefit. After fitting model M, we predict for each participant survival at time $t_{*}$ under treatment and control, that is, ${\hat{S}}_{\mathrm{iM}}^{1}\left(t_{*}\right)$ and ${\hat{S}}_{\mathrm{iM}}^{0}\left(t_{*}\right)$ respectively. Then, we transform these two probabilities using the complementary log–log transformation into log‐cumulative hazards, that is, $\log\left(-\log\left({\hat{S}}_{\mathrm{iM}}^{0}\left(t_{*}\right)\right)\right)$ and $\log\left(-\log\left({\hat{S}}_{\mathrm{iM}}^{1}\left(t_{*}\right)\right)\right)$. Subsequently, we fit two auxiliary Cox models, one for each treatment group, with sole covariate the corresponding estimated log‐cumulative hazard, modeled flexibly via splines. We then use the two fitted auxiliary models on all patients in the dataset, to predict survival under treatment and control at time $t_{*}$, conditional on their log‐cumulative hazards predicted by the model M. The difference between the two predictions will serve as the “observed” benefit in survival at time $t_{*}$. We finally compare this quantity with the survival benefit directly predicted by the model M as the difference between ${\hat{S}}_{\mathrm{iM}}^{1}\left(t_{*}\right)$ and ${\hat{S}}_{\mathrm{iM}}^{0}\left(t_{*}\right)$ and plot smooth calibration curves. For internal validation, the whole procedure needs to be performed using out‐of‐sample data, that is, after k‐fold cross‐validation. The method can be repeated for any choice of $t_{*}$. A limitation of this approach is that assumes proportional hazards on the auxiliary models (although more advanced models could be used); also, the “observed” benefit is not exactly observed, but relies on the auxiliary models.

### 4.4 Decision Accuracy

We previously proposed population benefit (PB) as a measure for decision accuracy [16]. The quantity of interest is the population‐level effect of using model M to decide whether to treat participants. Using model M we predict participant‐level treatment benefit ${\hat{B}}_{\mathrm{iM}}$ (following any of the definitions in Section 2.2, for example, ${\hat{B}}_{\mathrm{iM}}$ can be $B_{i}^{\mathrm{SP}}$, $B_{i}^{\mathrm{RST}}$ etc.). Then, we compare ${\hat{B}}_{\mathrm{iM}}$ with a threshold; if ${\hat{B}}_{\mathrm{iM}}>B_{\mathrm{Th}}$, the model suggests we should treat. In what follows we will set $B_{\mathrm{Th}}=0$ for simplicity. We are interested in whether this decision rule, that is, using model M to decide, leads to any benefit in terms of survival versus using reference decision strategies. In other words, we want to assess the causal effect of the intervention “use the model to decide how to treat” versus a control intervention.

The first family of estimands of interest [16] regards comparing outcomes after using the model to decide how to treat versus following the opposite of the model suggestions. To quantify, we can use again any of the link functions of Section 2.2:

(1) $$\begin{array}{cc} {\mathrm{PB}}_{M} & =E\left(g\left(S_{i}\right)|\text{treat according to model}\;M\right)\\ & \;-E\left(g\left(S_{i}\right)|\text{treat with the opposite of model}\;M\right) \end{array}$$

For example, the general definition of Equation (1) leads to the following specific definition if we focus on benefit in survival probability at time $t_{*}$:

(2) $$\begin{array}{cc} {\mathrm{PB}}_{M}^{\mathrm{SP}}\left(t_{*}\right) & =E\left(S_{i}\left(t_{*}\right)|\text{treat according to model}\;M\right)\\ & \;-E\left(S_{i}\left(t_{*}\right)|\text{treat with}\;t\mathrm{he}\;\text{opposite of model}\;M\right) \end{array}$$

**FIGURE 1:**
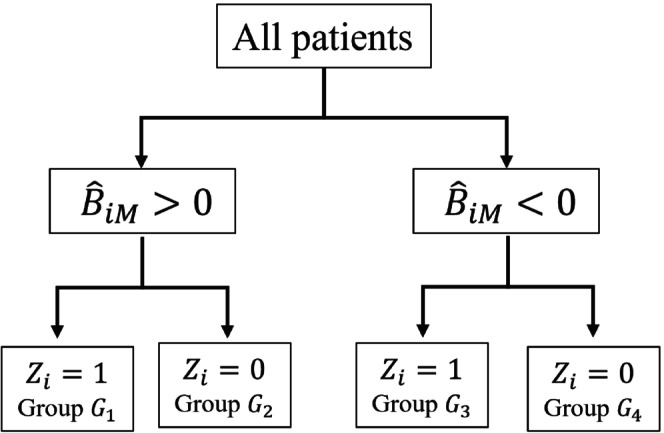
Assigning participants into groups according to the predicted treatment benefit (${\hat{B}}_{\mathrm{iM}}$) from a model (M), and treatment actually received ($Z_{i}=0/1$). Groups $G_{1}$ and $G_{4}$ include participants receiving the optimal treatment according to the model, $G_{2}$ and $G_{3}$ participants receiving the suboptimal.

Simply put, ${\mathrm{PB}}_{M}^{\mathrm{SP}}\left(t_{*}\right)$ measures the increase or reduction in average survival probability at time $t_{*}$ when following the model's suggestions compared to doing the opposite of what the model suggests. For estimation, we split participants according to ${\hat{B}}_{\mathrm{iM}}$ and the treatment each participant received into four groups (Figure 1). Participants in $G_{1}$ and $G_{4}$ received their optimal treatment, participants in $G_{2}$ and $G_{3}$ their suboptimal treatment, according to model M. We then compare outcomes in the combined $G_{1}UG_{4}$ group to the combined $G_{2}UG_{3}$ group using any of the treatment benefit definitions described in Section 2.2. Crucially, due to randomization there is no confounding when comparing outcomes in $G_{1}$ versus $G_{2}$ or when comparing $G_{3}$ versus $G_{4}$; thus, the comparison of $G_{1}UG_{4}$ versus $G_{2}UG_{3}$ is also unconfounded. This rests on the fact that ${\hat{B}}_{\mathrm{iM}}$ is obtained using out‐of‐sample methods (Section 4.1), so it is in essence a baseline variable in our dataset. This means that ${\hat{B}}_{\mathrm{iM}}$ is calculated without using any post‐randomization information from participant i. This allows us to split participants in groups according to ${\hat{B}}_{\mathrm{iM}}$ and treatment received, and interpret the observed difference in outcomes as causal. In other words, the group allocation for each participant according to ${\hat{B}}_{\mathrm{iM}}$ and treatment is not affected by the participant's own outcome, so that the effect is estimated unbiasedly.

For model M to be useful, ${\mathrm{PB}}_{M}$ needs to be larger than the average treatment effect (assuming again $B_{\mathrm{Th}}=0$). The average treatment effect corresponds to the PB of the simplest decision strategy, that is, one that treats all participants. This strategy suggests treating all participants or no‐one, depending on the sign of the average treatment effect. Thus, the following estimands are clinically relevant:

$$\begin{array}{cc} {\mathrm{PB}}_{\mathrm{MvC}} & =E\left(g\left(S_{i}\right)|\text{treat according to model}\;M\right)\\ & \;-E\left(g\left(S_{i}\right)|\text{treat}\;\mathrm{no}\;\mathrm{one}\right) \end{array}$$

$$\begin{array}{cc} {\mathrm{PB}}_{\mathrm{MvT}} & =E\left(g\left(S_{i}\right)|\text{treat according to model}\;M\right)\\ & \;-E\left(g\left(S_{i}\right)|\text{treat everyone}\right) \end{array}$$

If we focus on survival probability at a fixed time, these translate into:

$$\begin{array}{cc} PB_{\mathrm{MvC}}^{\mathrm{SP}}\left(t_{*}\right) & =E\left(S_{i}\left(t_{*}\right)|\text{treat according to model}\;M\right)\\ & \;-E\left(S_{i}\left(t_{*}\right)|Z_{i}=0\right) \end{array}$$

$$\begin{array}{cc} PB_{\mathrm{MvT}}^{\mathrm{SP}}\left(t_{*}\right) & =E\left(S_{i}\left(t_{*}\right)|\text{treat}\;ac\text{cording to model}\;M\right)\\ & \;-E\left(S_{i}\left(t_{*}\right)|Z_{i}=1\right) \end{array}$$

These two measures capture the benefit of personalizing treatment according to model M versus assigning the same treatment to everyone. To estimate ${\mathrm{PB}}_{\mathrm{MvT}}$, we compare survival in groups $G_{1}UG_{4}$ (participants treated according to the model) versus $G_{1}UG_{3}$ (participants receiving active treatment). In the Appendix A we show how to obtain standard errors of this quantity. Of note, although ${\mathrm{PB}}_{\mathrm{MvC}}$ and ${\mathrm{PB}}_{\mathrm{MvT}}$ are more easily interpretable, when it comes to comparing models all these performance measures rank models in the same way [16].

A useful property of population benefit is that it can be estimated for any type of prediction model, even models only predicting benefit, but not survivals under treatment and control, and for any definition of treatment benefit. To clarify this issue better, let us assume that the model we used is a simple Cox proportional hazards model. The model cannot readily be used to estimate treatment benefit in scales other than HR, without making some extra assumptions about baseline hazard. However, we can use the model to split participants into groups as shown in Figure 1 and then calculate ${\mathrm{PB}}_{M}$ using any measure of benefit described in Section 2.2.

The methods described in this paragraph are similar to approaches proposed by Nguyen et al. [40] and by Kapelner et al. [21] For a comparison between methods for measuring decision accuracy and methods for decision analysis, we refer the readers to our previous publication [16]; see also the Discussion section of the current paper.

## 5 Results

### 5.1 Simulated Dataset

#### 5.1.1 Fitting the Model and Making Predictions

**FIGURE 2:**
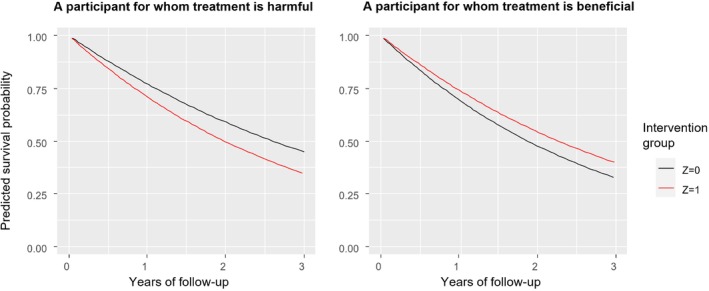
Predicted survival curves for two random participants in the simulated dataset, for treatment (*Z* = 1) and control (*Z* = 0). The predicted benefit in survival is the vertical distance between the two prediction curves at each time. Benefit in expected survival time corresponds to the difference in the area under the predicted survival curves, after extrapolating them to infinite times. Benefit in expected restricted survival time until time t* corresponds to the difference in the area under the two curves up to t*. Benefit in median survival corresponds to the horizontal distance between the two predicted curves at 50% survival. *Z* = 0 (*Z* = 1) denotes the control (treatment) group. According to the model, the treatment is beneficial for the participant in the right panel, but not for the one in the left.

We fit the model using all data. Figure 2 illustrates the predicted survival curves from the model for two random participants in the dataset. This figure serves to illustrate the heterogeneity of predicted treatment effects. Specifically, the participant of the left panel had covariates $x_{1}=-0.8,x_{2}=-0.9,x_{3}=1,x_{4}=0$. We can predict treatment benefit at the participant‐level using any of the definitions provided in Section 2.2, and for any time point. For this participant, the benefit in survival probability in 2 years is ${\hat{B}}_{i}^{\mathrm{SP}}\left(2\;\text{years}\right)=-9.3\%$; this corresponds to the vertical distance between the two curves at 2 years. The negative sign signifies that, according to the model, treating this individual would negatively impact their survival. The benefit in expected survival time is −0.9 years, corresponding to the difference in the area under the two survival curves after extrapolating them to infinity. The benefit in expected restricted survival time at 2 years is −0.1 years, corresponding to the difference in the area under the two curves up to year 2. Benefit in median survival is −0.6 years, corresponding to the horizontal distance between the two predicted curves at 50% survival (*y*‐axis in Figure 2). Conversely, for the participant of the right panel, treatment would be beneficial according to the model.

#### 5.1.2 Apparent Performance

Predictions of survival across all participants can be used to calculate the apparent performance for benefit in the original training sample. This assessment, as previously noted, runs the risk of being optimistic and is thus largely useless in practice. It can only be helpful as a sanity check, that is, to check procedures and software code. We show it to make the comparison with the out‐of‐sample performance presented below. In what follows, we outline results. Some additional details are provided in the Appendix A.

According to the model, 81% of the participants should be treated, and 19% should not. Next, we calculated all measures of performance described in Section 4.

For discrimination, we estimated C‐for‐benefit after matching participants one‐to‐one according to their predicted benefit in terms of survival at 2 years, that is, ${\hat{B}}_{i}^{\mathrm{SP}}\left(2\;\text{years}\right)$. Because the treatment groups were of unequal size, some of the participants in the larger group would have been excluded in a matching procedure. To ensure that all participants contributed to the analysis, we repeated the matching 100 times, with different participants being excluded each time. Despite the model being correctly specified and the sample size being relatively large (2000 patients, 979 events), we found that C‐for‐benefit was estimated at 0.54 [95% Confidence Interval 0.51–0.57]. Of note, this CI ignores uncertainty in the matching procedure. When re‐calculating C‐for‐benefit after matching participants one‐to‐one according to their covariates using propensity score matching, repeating 100 times, we got 0.55 [0.52; 0.58].

For calibration for benefit, we compared the Kaplan–Meier curve for each treatment group with the predicted survival from the model. The plot (shown in the Appendix A) showed very good agreement, as expected. Next, we split participants into groups as per Figure 1 and compared predicted and observed benefit at the group level. We did this using both the benefit in survival probability in 2 years (${\hat{B}}_{i}^{\mathrm{Su}}\left(2\;\text{years}\right)$) and the benefit in restricted mean survival time in 2 years (${\hat{B}}_{i}^{\mathrm{RST}}\left(2\;\text{years}\right)$). Results are shown in the Appendix A, showing perfect apparent calibration. Finally, we created a calibration‐for‐benefit curve following the model‐based approach. The plot is provided in the Appendix A, showing perfect calibration for benefit. Mean bias of benefit was low: using survival probability and RMST at 2 years ${\text{Bias}}_{M}$ was 0.7% and 0.004 years respectively.

We turned to decision accuracy. The population benefit of the model at 2 years was estimated at ${\hat{\mathrm{PB}}}_{M}^{\mathrm{SP}}\left(2\;\text{years}\right)$ = 13% [8%; 18%]. When compared to treating everyone we found ${\hat{\mathrm{PB}}}_{\mathrm{MvT}}^{\mathrm{SP}}\left(2\;\text{years}\right)$ = 2% [0%; 4%], while compared to treating no‐one we found ${\hat{\mathrm{PB}}}_{\mathrm{MvC}}^{\mathrm{SP}}\left(2\;\text{years}\right)$ = 12% [7%; 16%]. Interestingly, among the subpopulation of patients where the treatment predicted negative benefit, treatment effect was −10% [−21%; 1]. More results are shown in the Appendix A.2.

#### 5.1.3 Using 10‐Fold Cross‐Validation for Assessing Performance

We assessed the model's out‐of‐sample predictive performance using a 10‐fold CV procedure to obtain predictions of survival probabilities over time for each participant under treatment and control. These were then used to calculate all performance measures.

For discrimination for benefit, we estimated C‐for‐benefit after matching participants one‐to‐one according to predicted benefit, repeating 100 times. C‐for‐benefit was estimated at 0.53 [0.50; 0.56]. We repeated after matching for participant covariates and found 0.54 [0.51; 0.57]. These estimates were slightly lower than the ones from apparent performance.

**FIGURE 3:**
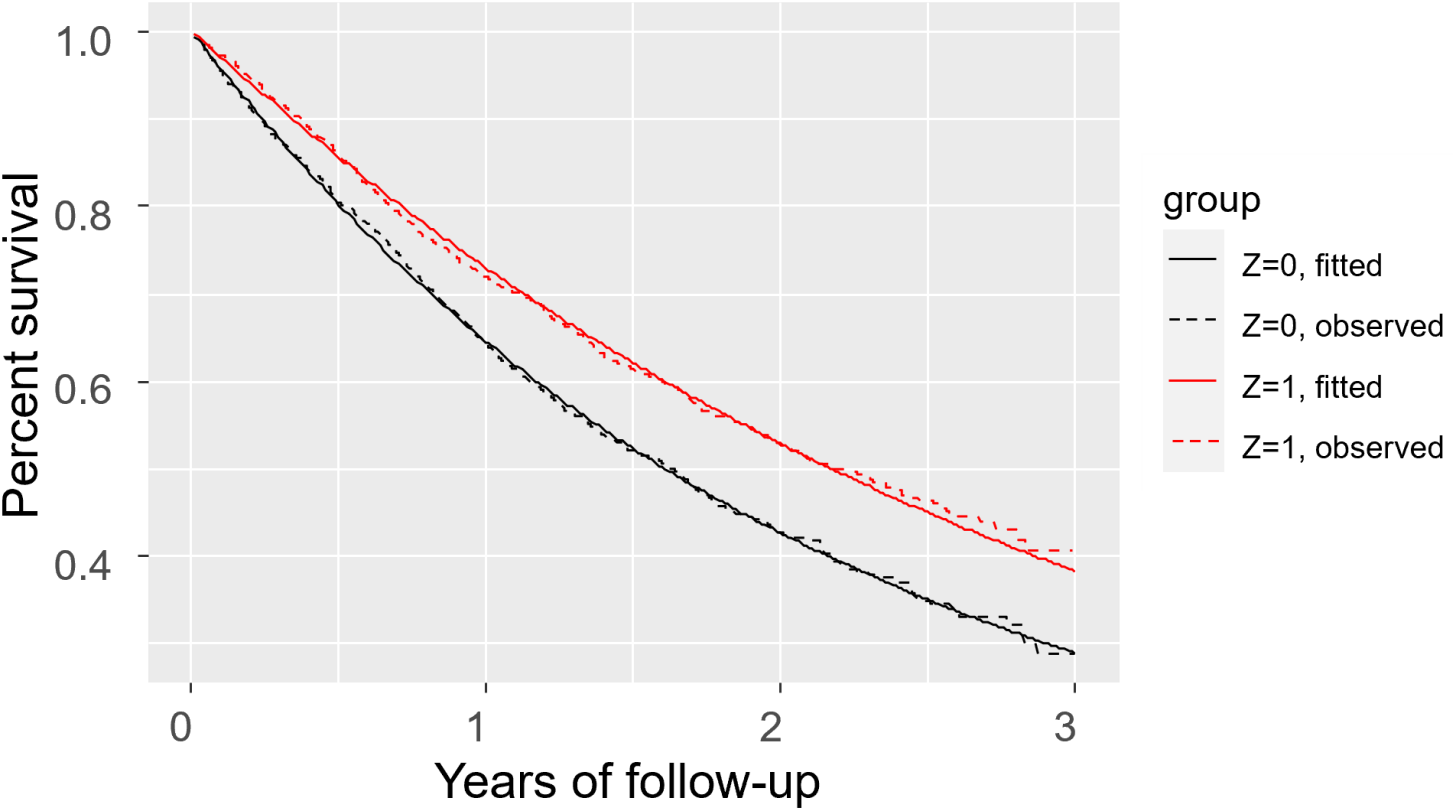
Observed survival as Kaplan–Meier curves (dotted lines) versus model predictions, for the simulated example. *Z* = 0 (*Z* = 1) denotes the control (treatment) group. The graph is based on out‐of‐sample predictions following a 10‐fold cross‐validation.

**FIGURE 4:**
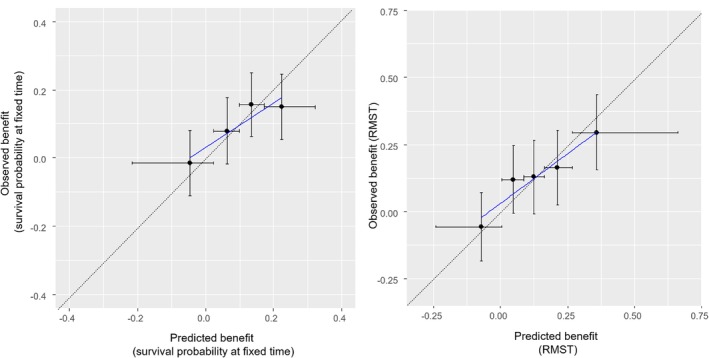
Calibration‐for‐benefit plots for the simulated dataset. The dataset is split into five groups according to predicted benefit in survival probability at 2 years (left panel) and according to restricted mean survival time (RMST) in 2 years (right panel). Within each group, we compare the mean predicted across participants with the observed one. Graphs are based on out‐of‐sample predictions, following a 10‐fold cross‐validation. Error bars on the *y*‐axis show upper and lower 95% confidence intervals. Error bars on the *x*‐axis show the range of predicted benefit within each group.

**FIGURE 5:**
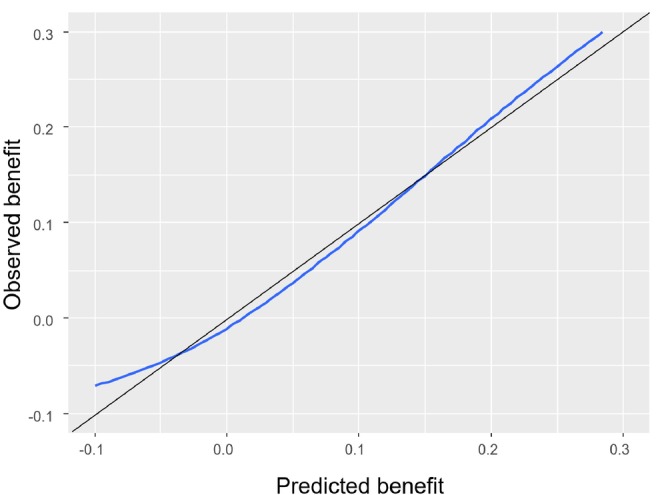
Smooth calibration‐for‐benefit curve for the simulated dataset using restricted cubic splines in conjunction with a Cox proportional hazards model.

For assessing calibration‐for‐benefit, we first compared the Kaplan–Meier curves for each treatment group with the predicted survival from the models. Figure 3 summarizes the data (dotted lines, Kaplan–Meier curves) and model predictions (thick lines) across the two intervention groups, and shows that the model was well calibrated on average. Next, we assessed calibration‐for‐benefit after grouping into five groups. The left panel of Figure 4 used ${\hat{B}}_{i}^{\mathrm{SP}}\left(2\;\text{years}\right)$ to group participants and showed some overestimation of benefit, as the slope of the calibration line was below 1, estimated at 0.63 [0.20; 1.06]. Mean bias was 0.0% and RMSE was 4.4%. At the right panel of Figure 4 we provide the calibration for benefit line using ${\hat{B}}_{i}^{\mathrm{RST}}\left(2\;\text{years}\right)$. Slope was 0.70 [0.34; 1.04], mean bias was 0.0, RMSE 0.5 years. Finally, the calibration curve using a spline model with three knots is shown in Figure 5. Mean bias of benefit using survival probability and RMST at 2 years (${\text{Bias}}_{M}$) was 0.6% and 0.004 years respectively.

Next, we turned to decision accuracy. Population benefit at 2 years was estimated at ${\hat{\mathrm{PB}}}_{M}^{\mathrm{SP}}\left(2\;\text{years}\right)$ = 10% [5%; 15%]. Compared to the strategy to treat all, the model showed no evidence for population benefit, ${\hat{\mathrm{PB}}}_{\mathrm{MvT}}^{\mathrm{SP}}\left(2\;\text{years}\right)$ = 0% [−2%; 3%]. This suggests that using the model would not lead to difference of the outcome at the population level, as compared to just treating everyone. Compared to the strategy of treating no one, the model showed population benefit ${\hat{\mathrm{PB}}}_{\mathrm{MvC}}^{\mathrm{SP}}\left(2\;\text{years}\right)$ = 10% [5%; 14%]. Among the subpopulation of patients where the model predicted negative benefit, treatment effect was −2% [−13%; 9%], showing that the model could not accurately identify patients that should avoid treatment. More results are shown in the Appendix A.2.

In summary, we found that for some measures, the out‐of‐sample performance of the model was somewhat lower than the apparent performance, thus possibly removing optimism. Model discrimination was low, while decision accuracy measures did not show substantial benefits at the population level when using the model. However, the model identified patients that would benefit substantially from treatment, as shown in Figures 4 and 5 (i.e., groups on the top right corner). This might be useful in case prioritizing treatment was of interest.

### 5.2 The OPERAM Trial

We used the data described in Section 3.2 to develop two competing models for predicting treatment effects at the patient level. To illustrate the methods, we used a statistical and a machine learning model. First, we used a Weibull survival model with linear terms for all covariates described in Section 3.2, the intervention indicator, and interaction terms between all covariates and the intervention. Second, we used a random forest model with the same covariates and interactions as above. This approach is termed “S‐learner” (single learner) approach, that is, to include the intervention as just another covariate in the model [41]. In what follows, we show performance after a 10‐fold cross‐validation.

For discrimination for benefit, we estimated C‐for‐benefit after matching patients one‐to‐one according to the predicted benefit at survival probability ${\hat{B}}_{i}^{\mathrm{SP}}\left(1\;\text{year}\right)$ from the models, repeating 100 times. C‐for‐benefit was estimated at 0.55 [0.52; 0.58] for Weibull, 0.53 [0.50; 0.56] for random forest.

**FIGURE 6:**
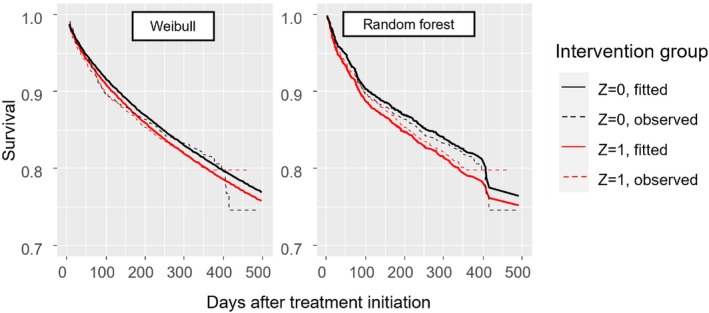
Observed survival as Kaplan–Meier curves (dotted lines) versus model predictions (left panel: Weibull model; right panel: Random forest), for the OPERAM dataset. The graph is based on out‐of‐sample predictions, following a 10‐fold cross‐validation. *Z* = 0 (*Z* = 1) denotes the control (active) intervention.

**FIGURE 7:**
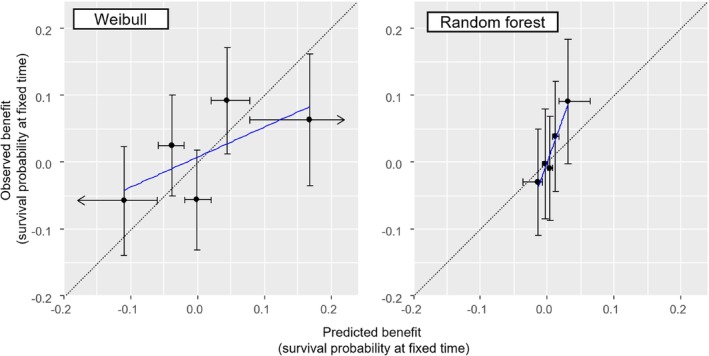
Calibration‐for‐benefit plots for the OPERAM dataset. The dataset is split into five groups according to predicted benefit in survival probability at 1 year (left panel: Weibull model; right panel: Random forest). Within each group, we compare the mean predicted across patients with the observed one. Graphs are based on out‐of‐sample predictions, following a 10‐fold cross‐validation. Error bars on the *y*‐axis show upper and lower 95% confidence intervals. Error bars on the *x*‐axis show the range of predicted benefit within each group.

**FIGURE 8:**
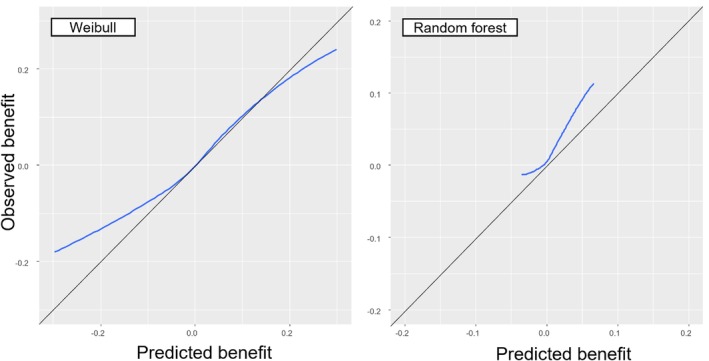
Smooth calibration‐for‐benefit curve for the OPERAM dataset using restricted cubic splines in conjunction with a Cox proportional hazards model. Left panel: Weibull. Right panel: Random forest. Graphs are based on out‐of‐sample predictions, following a 10‐fold cross‐validation.

To assess calibration‐for‐benefit, we compared the Kaplan–Meier curves for each intervention group with the predicted survival from the two models. Figure 6 shows that both models accurately predict survival between 200 and 400 days, capturing the (small) differences between the groups. Next, we assessed calibration‐for‐benefit after grouping into five groups according to ${\hat{B}}_{i}^{\mathrm{Su}}\left(1\;\text{year}\right)$. The left panel of Figure 7 shows Weibull, the right shows random forest. For Weibull we see evidence of overestimation of treatment effects, where the slope of the line was estimated at 0.45 [−0.43; 1.34], mean bias at 0.0% and RMSE at 7.0%. Conversely, for random forest we see clear signs of underestimation of effects, slope 2.77 [1.52; 4.01], mean bias = 1%, and RMSE 3%. When we switched to RMST we saw the same picture (results not shown). Finally, the calibration line using a spline with three knots is shown in Figure 8, where we see a good agreement with Figure 7, although the two analyses follow different approaches.

Finally, we turned to decision accuracy. Population benefit at 1 year was estimated at ${\hat{\mathrm{PB}}}_{M}^{\mathrm{SP}}\left(1\;\text{year}\right)$ = 6% [2%; 10%] for Weibull, 4% [0%; 7%] for random forest. Compared to the strategy to use the intervention for all, both models showed limited evidence for population benefit, ${\hat{\mathrm{PB}}}_{\mathrm{MvT}}^{\mathrm{SP}}\left(1\;\text{year}\right)$ = 2% [−2%; 7%] for Weibull, 1% [−1%, 3%] for random forests. Among the subpopulation of patients where the Weibull model predicted negative benefit, the observed intervention effect was −4% [−9%; 1%], showing weak evidence that the model could possibly identify patients that should avoid intervention. For random forest it was −2% [−8%; 4%], showing that the model completely failed to identify such patients. Results from estimating decision accuracy for benefit in RMST led to similar conclusions (not shown here).

Overall, we found that neither of the two models yielded good performance in predicting treatment effects in general on all‐cause mortality, and particularly in identifying patients for whom the intervention would be harmful. There was some (weak) evidence that the models could identify patients benefiting to a greater extent from the intervention, as shown at the top right corner in Figures 7 and 8 (although estimates tended to be miscalibrated).

## 6 Software Code

We provide code for implementing the methods is R in https://github.com/oremiou/performance‐for‐treatment‐benefit‐survival.The following packages were employed: ggplot2 [42], ggfortify [43], survival [44], survRM2 [45], Hmisc [46], Matching [47], rms [48], pec [49], flux [50], randomForestSRC [51].

## 7 Discussion

In this paper, we described new methods and measures for assessing the performance of models that predict treatment effect at the participant‐level, for the case of a survival (time‐to‐event) outcome. Our methods can be used for both statistical and machine‐learning models. Further, all methods can be used either for internal validation of a model (i.e., following model development) or in an external validation study (i.e., when researchers want to validate an existing model using new data). Specialized methods such as the ones we presented are required to assess the performance of a model predicting benefit from treatment. This is because minimizing the error of outcome predictions does not necessarily minimize the error of the benefit predictions [14, 15, 16]. In other words, it is not enough to assess the performance of predictions for outcomes in both the treatment and the control groups; a model might perform adequately for this, but at the same time, it may completely fail to predict benefit. In Section 5 of the Appendix, we provide a simulated example where a prediction algorithm performs well in predicting survival in both the treatment and the control groups according to performance metrics focused on outcomes; however, the model cannot predict treatment benefit, and only methods that focus on benefit such as the ones we described can identify the problem. Moreover, there are models that only predict treatment benefit without providing outcome predictions; in such cases, performance measures focusing on outcomes cannot be used.

The current article extends the scope of our previous work that presented methods for assessing models predicting personalized treatment effects for continuous and binary outcomes [16, 27]. A survival outcome can always be dichotomized at a target time point (e.g., at day 30 post intervention), and our previously described methods for binary outcomes could be used for survival outcomes as well. This approach, however, would lead to a loss of statistical power [52, 53], would be dependent on the choice of the target time point, and would not adequately handle censoring before the targeted time point.

Here we described a range of methods and metrics for assessing discrimination‐for‐benefit, calibration‐for‐benefit, and decision accuracy. The latter is a dimension of performance that combines discrimination and calibration, and aims to summarize the population‐level benefit of using a model for personalized effects to make treatment decisions [16]. An advantage of decision benefit is that it is easy to understand (i.e., “*what would be the population‐level benefit if I used the model to make treatment decisions as opposed to just treating everyone*?”). However, if the model is useful for only a small number of participants (i.e., in identifying the few participants that will benefit from treatment), decision accuracy measures may show that the model has little effect at the population level, thus diluting its potential usefulness, while statistical power may be too low. Another limitation of this approach is that it only focuses on assessing the predictive performance of a model for benefit for a single outcome. In real‐world decision making, analysis methods such as a cost–benefit analysis [26], a decision curve analysis [6, 54], or a multiple criteria decision analysis (MCDA [55]) can be used to assess the impact of a model while taking into account multiple outcomes, that is, benefits and harms, but also costs and general disutility associated with interventions. Such analyses can be used to establish the clinical and wider benefits of using a prediction model in decision making, and require additional input, for example, on costs or preferences. Conversely, we hereby focus solely on methods for measuring the predictive performance of models for treatment benefit.

A further limitation of our paper relates to measures for discrimination‐for‐benefit. Here we only described the previously proposed C‐for‐benefit [19]. One problem with this metric is that the value it produces may be hard to interpret; for example, depending on the context, a C‐statistic of 0.60 might be considered relatively low for prognostic discrimination but might be high for benefit. Moreover, C‐for‐benefit shares some of the deficiencies of the usual C‐index for usual survival models, as summarized by Hartman et al. [38] For example, when calculating C‐for‐benefit, the magnitude of observed or predicted benefit in matched pairs is not taken into account. This, of course, leads to a loss of information and may partly explain the low values we see in practice. However, this is the cost of having a pure discrimination measure, that takes only rank ordering into account; in other words, it is a feature of the discrimination estimand, and not the estimator itself. Another important argument against C‐for‐benefit is that, as Hartman et al. [38] discuss for the regular C‐index, it rarely addresses real clinical questions. For example, a clinician might be interested in the expected benefit of prescribing a new drug to patient X and whether a prediction model performs well with respect to this task (i.e., a question related to calibration for benefit). The question of whether the model accurately identifies whether patient X will benefit more from the drug than patient Y does (i.e., a question related to discrimination for benefit) is much less likely to be clinically relevant. Despite these deficiencies, Van Klaveren et al. [19] illustrated that the metric might be useful to compare competing models, while several extensions have been recently proposed [20, 27, 56]. However, Xia et al. [39] showed that even using the correct model might lead to C‐for‐benefit values below 0.5, under certain circumstances. Taking all this into account, we recommend that the C‐for‐benefit should be used with caution and always in conjunction with measures for calibration for benefit.

An anonymous reviewer suggested that in order to decide on whether to treat or not, we should compare the lower end of the 95% confidence interval (CI) of the prediction with the threshold for decision. However, as Vickers et al. [57] suggested, uncertainty measures such as *p*‐values or CIs are of questionable value when assessing prediction models, and that “When we are forced into a decision, we should choose the option with the highest expected utility, irrespective of *p* values or uncertainty.” In addition, for some type of survival models it may hard to obtain CIs, often requiring computationally intensive methods such as bootstrapping [58]. For these reasons, our metrics for decision accuracy are based on the individual‐level predicted benefit, without taking into account the uncertainty around it.

In our examples, we focused on the internal validation case, and illustrated our methods using data from a large trial on a pharmacotherapy optimization intervention (OPERAM) [36]. For illustrative purposes, we developed two competing models for predicting survival benefit using this trial's data: a Weibull regression model and a random forest. Then, we used existing as well as our newly developed methods to assess their respective performance in an internal validation procedure. We illustrated how model performance can be assessed via a 10‐fold cross‐validation approach. In real applications, we would repeat the k‐fold analysis multiple times, to ensure stability of results. Of note, with this approach, all folds stem from the same data generating mechanism. Instead of k‐fold cross‐validation, we could have used a leave‐one‐out method. Specifically, in the OPERAM trial, data were collected in four sites. Using this information, we could have performed an internal‐external cross‐validation [59], where we would develop the model in three sites and test in the fourth, cycling through all sites. This procedure might also inform us about the heterogeneity of model performance when used in a different setting. However, we did not pursue this here, for simplicity. Our analysis found generally unsatisfactory performance for both the Weibull and the random forest models. Nonetheless, there was some evidence that the models could identify patients for whom the intervention could be particularly effective. This could inform the design of a future RCT, that is, where the intervention could only be tested on such patients.

Generally, as we saw in our example analyses, it is very difficult to predict benefit at the participant‐level. Very large datasets are required for such endeavors. In this paper we focused on randomized data from a single RCT. RCTs, however, are typically powered to detect average effects, and are underpowered to detect participant‐level effects; as previously noted, “even if an optimal personalized medicine rule can provide substantial gains it may be difficult to estimate this rule with few subjects” [60]. Thus, an extension to individual participant data (IPD) meta‐analysis [61] or even better IPD network meta‐analysis (for the case of multiple treatments) is warranted [62, 63]. Recently, methods have been proposed for the case of observational data [64, 65]. Observational data have many limitations for such analyses—primarily confounding—but may assemble large number of individuals. An interesting project would be the extension of our methods for the case of observational studies. Moreover, in the introduction of the current paper we briefly outlined a large array of previously proposed methods for assessing the performance of models predicting personalized benefit. Broadly, these methods either extend traditional metrics for clinical prediction models or evaluate methods for optimal treatment assignment. However, it is currently unclear how all these methods compare with each other as well as to the new methods we proposed. Thus, an idea for future research would be catalogue, review, summarize, and contrast in simulations all available methods. Such a review could clarify the overlap and complementarity of these models, identify which ones apply to survival models, and would be useful in guiding practical applications as to what measures are more clinically relevant, sensitive, and reliable. Another interesting direction would be to aim at developing models that maximize performance measures related to treatment benefit, rather than absolute outcome prediction. For example, when developing penalized models, the penalty parameter could be chosen in a k‐fold cross‐validation approach that maximizes decision accuracy measures. One other possible stream of future research would be to extend alternative metrics proposed for usual prediction modeling (such as the index of prediction accuracy, that is, a scaled version of the Brier score) to cover the case of treatment benefit [66].

Finally, we would like to make a plea for the need for external validation of models predicting individual‐level treatment effects. External validation has been recognized to be a prerequisite for usual clinical prediction models [59], before they are applied in practice. We deem that it is equally, if not more important, for models aiming at treatment effects. In this work, we have provided tools for such validation.

To summarize, we have presented a range of methods for assessing the performance of models that aim to predict the effects of treatment at the level of the individual. Our methods can be used when developing a new model or when validating an existing, pre‐developed model and are applicable to assessing statistical and machine learning models.

## Conflicts of Interest

The authors declare no conflicts of interest.

## Figures and Tables

**FIGURE A1:**
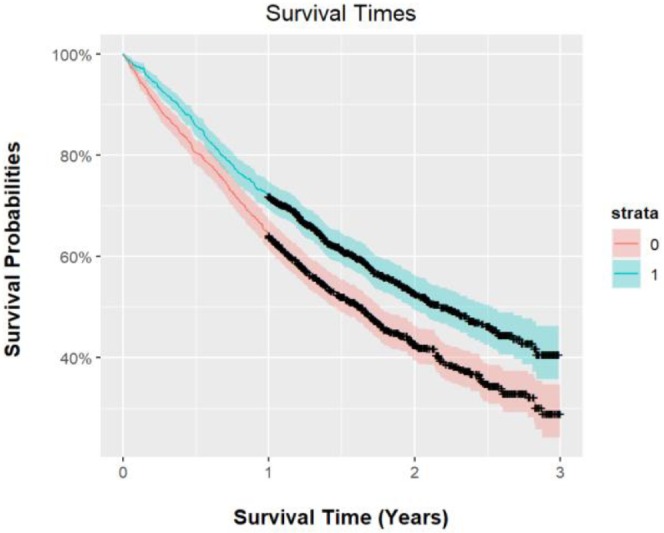
Kaplan–Meier curves for the two treatment groups in the simulated dataset. Crosses in the curves indicate censoring.

**FIGURE A2:**
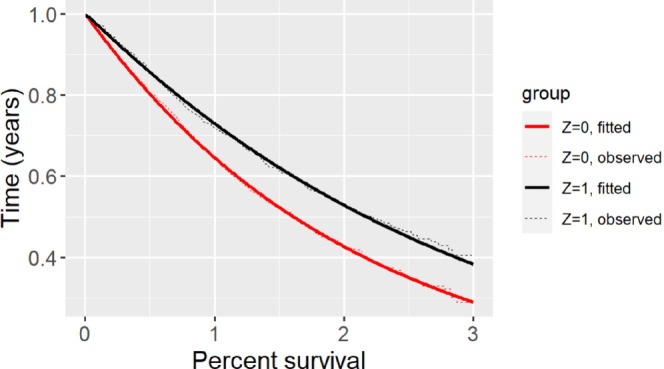
Observed survival as Kaplan–Meier curves (dotted lines) versus model predictions for the simulated example. *Z* = 0 (*Z* = 1) denotes the control (treatment) group. This graph shows apparent model performance (i.e., testing and training dataset were the same).

**FIGURE A3:**
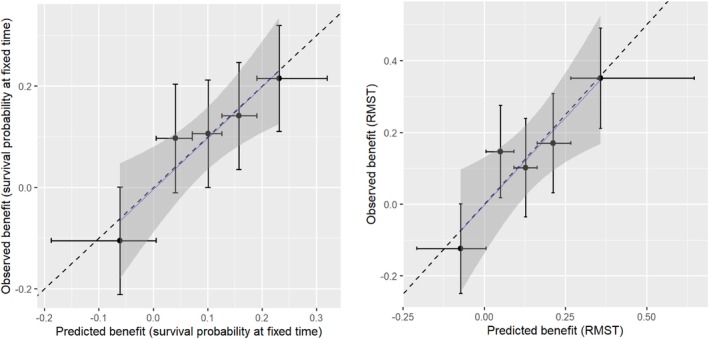
Graphical assessment of calibration‐for‐benefit. Left panel: Using predicted survival probability at 2 years. Right panel: Using restricted mean survival time (RMST) at 2 years. This graph shows apparent model performance (i.e., testing and training dataset was the same).

**FIGURE A4:**
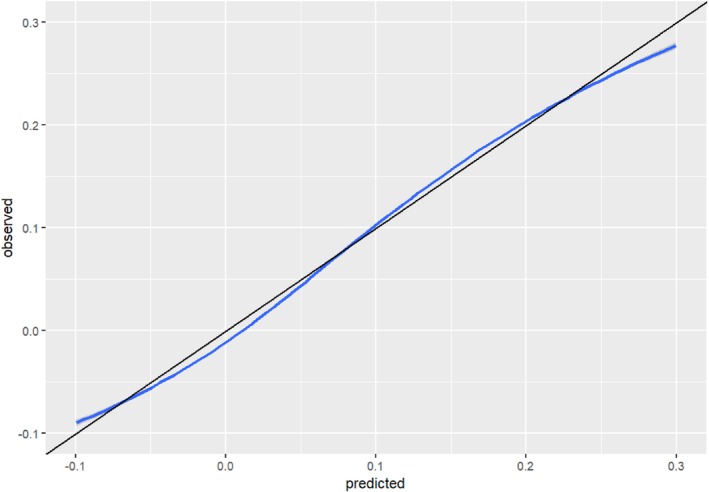
Model‐based smooth calibration curve for benefit.

## Data Availability

Data sharing is not applicable to this article as no new data were created or analyzed in this study.

## Appendix A Measuring the Performance of Survival Models to Personalize Treatment Choices

### Description of the Simulated Dataset

We simulated a dataset *N* = 2000 patients. For each patient i we generated four covariates ($x_{1i}\ldots x_{4i}$), of which two were continuous ($x_{1},x_{2}$), one categorical ($x_{3}$, with four categories), and one binary ($x_{4}$). To generate these covariates, we started by drawing 4‐vectors from a multivariate normal distribution with mean (0, 0, 0, 0) and variance–covariance matrix with an auto‐regressive form:

$$\sum =\left(\begin{array}{llll} 1 & 0.5 & 0.5^{2} & 0.5^{3}\\ 0.5 & 1 & 0.5 & 0.5^{2}\\ 0.5^{2} & 0.5 & 1 & 0.5\\ 0.5^{3} & 0.5^{2} & 0.5 & 1 \end{array}\right)$$

The first two elements of each 4‐vector corresponded to $x_{1},x_{2}$. We transformed the third element to $x_{3}$, a categorical variable with four categories (*A*, *B*, *C*, *D*) by using cutoffs at −1, 1, and 2 (i.e., so that a value of −5 would correspond to category *A*, a value of 0.5 to category *B*, etc.). We transformed $x_{4}$ to a binary variable by dichotomizing the fourth element of each 4‐vector at 0. Then, we generated the treatment indicator $Z_{i}$ by drawing from a Bernoulli distribution with 50% probability. Next, we calculated the patient‐level event rate as a function of covariates and treatments. More specifically, we set:

$$\begin{array}{cc} \mathrm{rat}e_{i} & =\exp\left(-1+0.3\;x_{1i}+0.\;3\;x_{2i}+0.2\;\left(x_{3i}==B\right)-0.2\;\left(x_{3i}==C\right)\right.\\ & \;\left.+0.1\;\left(x_{3i}==D\right)-0.1\left(x_{4i}==1\right)\right)+Z_{i}\left.-0.1-0.1\;x_{1i}-0.2\;x_{2i}\right)\\ & \;\left.+0.2\;(x_{3i}==B\right)-0.2\;\left(x_{4i}==1\right)) \end{array}$$

Then, for each patient we generated the survival time by drawing from an exponential distribution given the patient‐specific event rate. Finally, we generated censoring time by drawing from a uniform distribution as U(1,3). If censoring time was smaller than the survival time, the final status of the patient was set to 0; otherwise to 1.

Of the 2000 patients, 973 were randomized to control and 1027 to treatment. A total of 1021 patients had the event, and 979 were censored before they experienced the event. The median follow‐up time was 1.3 years. For 1421 patients (71%), the true event rates were larger in control rather than treatment, that is, for these patients, treatment benefit was positive.

Below we show the Kaplan–Meier curve for the two treatment groups (Figure A1).

We used this dataset to fit a Weibull model with all covariates and all treatment‐covariate interactions. The estimated coefficients from the model are as follows:

Coefficients:

**Table:**

(Intercept)	x1	x2	x32	x33	x34	x41
0.85065641	‐0.28471011	‐0.26237802	‐0.11424594	0.19829314	‐0.10744863	0.15997313
tr1	x1:tr1	x2:tr1	x32:tr1	x33:tr1	x34:tr1	x41:tr1
‐0.01698614	0.12804934	0.16866607	0.33796554	0.17074321	0.24031468	0.03085145

### Obtaining Standard Errors for Measures of Population Benefit

In Section 4.4 of the main paper, we introduced measures of population benefit:

$$B_{\mathrm{MvC}}^{\mathrm{SP}}\left(t_{*}\right)=E\left(S_{i}\left(t_{*}\right)|\text{treat acco}r\text{ding to model}\;M\right)-E\left(S_{i}\left(t_{*}\right)|Z_{i}=0\right)$$

$$PB_{\mathrm{MvT}}^{\mathrm{SP}}\left(t_{*}\right)=E\left(S_{i}\left(t_{*}\right)|\text{treat according to model}\;M\right)-E\left(S_{i}\left(t_{*}\right)|Z_{i}=1\right)$$

To estimate ${\mathrm{PB}}_{\mathrm{MvT}}$, we compare survival in groups $G_{1}UG_{4}$ (patients treated according to the model) versus $G_{1}UG_{3}$ (patients receiving treatment). Obtaining a standard error is not straightforward because the two groups are not independent, as they share $G_{1}$. One way to do it is to note that:

$$\begin{array}{cc} & E\left(s_{i}\left(t_{*}\right)|\text{treat according to model}\;M\right)\\ & \;=E\left(S_{i}\left(t_{*}\right)|G_{1}UG_{4}\right)=p\left({\hat{B}}_{M}>0\right)E\left(S_{i}\left(t_{*}\right)|G_{1}\right)\\ & \;+p\left({\hat{B}}_{M}<0\right)E\left(S_{i}\left(t_{*}\right)|G_{4}\right) \end{array}$$

Similarly

$$\begin{array}{cc} E\left(S_{i}\left(t_{*}\right)|Z_{i}=1\right) & =E\left(S_{i}\left(t_{*}\right)|G_{1}UG_{3}\right)=p\left({\hat{B}}_{M}>0\right)E\left(S_{i}\left(t_{*}\right)|G_{1}\right)\\ & \;+p\left({\hat{B}}_{M}<0\right)E\left(S_{i}\left(t_{*}\right)|G_{3}\right) \end{array}$$

Subtracting these two last equations, we obtain

$$PB_{\mathrm{MvT}}^{\mathrm{SP}}\left(t_{*}\right)=p\left({\hat{B}}_{M}<0\right)\left(E\left(S_{i}\left(t_{*}\right)|G_{4}\right)-E\left(S_{i}\left(t_{*}\right)|G_{3}\right)\right)$$

The standard error is now easy to estimate, using the following identity $\mathrm{Var}\left(\mathrm{XY}\right)=\mathrm{Var}\left(X\right)\mathrm{Var}\left(Y\right)+E{\left(X\right)}^{2}\mathrm{Var}\left(Y\right)+E{\left(Y\right)}^{2}\mathrm{Var}\left(X\right)$ when Cov(X,Y)=0

$$\begin{array}{cc} & \mathrm{Var}\left(PB_{\mathrm{MvT}}^{\mathrm{SP}}\left(t_{*}\right)\right)\\ & \;=\mathrm{Var}\left(p\left({\hat{B}}_{M}<0\right)\right)\left(\mathrm{Var}\left(S_{i}\left(t_{*}\right)|G_{4}\right)+\mathrm{Var}\left(S_{i}\left(t_{*}\right)|G_{3}\right)\right)\\ & \;+p{\left({\hat{B}}_{M}<0\right)}^{2}\left(\mathrm{Var}\left(S_{i}\left(t_{*}\right)|G_{4}\right)+\mathrm{Var}\left(S_{i}\left(t_{*}\right)|G_{3}\right)\right)\\ & \;+{\left(E\left(S_{i}\left(t_{*}\right)|G_{4}\right)-E\left(S_{i}\left(t_{*}\right)|G_{3}\right)\right)}^{2}\mathrm{Var}\left(p\left({\hat{B}}_{M}<0\right)\right)\\ & \;=\frac{p\left({\hat{B}}_{M}<0\right)\;p\left({\hat{B}}_{M}>0\right)}{N}\left(\mathrm{Var}\left(S_{i}\left(t_{*}\right)|G_{4}\right)+\mathrm{Var}\left(S_{i}\left(t_{*}\right)|G_{3}\right)\right)\\ & \;+p{\left({\hat{B}}_{M}<0\right)}^{2}\left(\mathrm{Var}\left(S_{i}\left(t_{*}\right)|G_{4}\right)+\mathrm{Var}\left(S_{i}\left(t_{*}\right)|G_{3}\right)\right)\\ & \;+{\left(E\left(S_{i}\left(t_{*}\right)|G_{4}\right)-E\left(S_{i}\left(t_{*}\right)|G_{3}\right)\right)}^{2}\frac{p\left({\hat{B}}_{M}<0\right)\;p\left({\hat{B}}_{M}>0\right)}{N} \end{array}$$

where, *N* is the total number of patients in the sample.

Similarly, to estimate ${\mathrm{PB}}_{\mathrm{MvC}}$, we compare survival in groups $G_{1}UG_{4}$ (patients treated according to the model) versus $G_{2}UG_{4}$ (patients receiving control), and the variance can be estimated as:

$$\begin{array}{cc} & \mathrm{Var}\left(PB_{\mathrm{MvC}}^{\mathrm{SP}}\left(t_{*}\right)\right)\\ & \;=\frac{p\left({\hat{B}}_{M}<0\right)\;p\left({\hat{B}}_{M}>0\right)}{N}\left(\mathrm{Var}\left(S_{i}\left(t_{*}\right)|G_{1}\right)+\mathrm{Var}\left(S_{i}\left(t_{*}\right)|G_{2}\right)\right)\\ & \;+p{\left({\hat{B}}_{M}>0\right)}^{2}\left(\mathrm{Var}\left(S_{i}\left(t_{*}\right)|G_{1}\right)+\mathrm{Var}\left(S_{i}\left(t_{*}\right)|G_{2}\right)\right)\\ & \;+{\left(E\left(S_{i}\left(t_{*}\right)|G_{1}\right)-E\left(S_{i}\left(t_{*}\right)|G_{2}\right)\right)}^{2}\frac{p\left({\hat{B}}_{M}<0\right)\;p\left({\hat{B}}_{M}>0\right)}{N} \end{array}$$

### Apparent Performance of Model Fit in the Simulated Dataset

In Figure A2 we show the Kaplan–Meier curves for each treatment group overlaid with the model predictions. Overall, we see very good agreement.

At the left panel of Figure A3 we show the calibration‐for‐benefit plot, produced after grouping patients into five groups according to their predicted survival probability at 2 years. The slope was 1.01 [0.40; 1.63] and the intercept was 0.00 [−0.09; 0.09]. The RMSE of group predictions was 3.4%, and mean bias was 0.3% across the five groups. At the right panel, we repeat this procedure, but this time splitting according to restricted mean survival time (RMST) at 2 years. The slope of the line was 0.98 [0.31; 1.65] and the intercept 0.00 [−0.14; 0.13]. The RMSE was 0.05 years, and mean bias across the groups was 0.01 years.

In Figure A4 we also show the model‐based plot for calibration for benefit. This uses a spline function with three knots.

In Table A1 we see results for decision accuracy. Although the model, at the population level, did not perform substantially better than the simple strategy of treating everyone, it was able to identify individuals for whom treatment would be harmful (as indicated by the negative values for $B_{\hat{B}<0}^{\mathrm{obs}}$). However, this is the apparent performance.

**TABLE A1:** Measures for decision accuracy, for the simulated dataset (apparent performance).

Estimand	Estimated value if treating…				Estimated population benefit
	No‐one	Everyone	With model	Against model	
Median survival	1.63 y [1.44; 1.76]	2.22 y [1.98; 2.53]	2.31 y [2.08; 2.61]	1.55 y [1.38; 1.69]	$PB_{M}$ = 0.78 y ${\mathrm{PB}}_{M}^{\left(1\right)}$ = 0.09 y $B_{{\hat{B}}_{M}<0}^{\mathrm{obs}}$ = −0.25 y
1‐year survival	64% [61%; 67%]	72% [69%; 75%]	73% [70%; 76%]	64% [61%; 67%]	$PB_{M}$ = 9% [5%; 13%] ${\mathrm{PB}}_{M}^{\left(1\right)}$ = 1% [−4%; 6%] $B_{{\hat{B}}_{M}<0}^{\mathrm{obs}}$ = −5% [−14%; 3%]
2‐year survival	43% [39%; 46%]	53% [49%; 56%]	54% [51%; 58%]	41% [38%; 45%]	$PB_{M}$ = 13% [8%; 18%] ${\mathrm{PB}}_{M}^{\left(1\right)}$ = 2% [−5%; 8%] $B_{{\hat{B}}_{M}<0}^{\mathrm{obs}}$ = −10% [−21%, 1%]
RMST to 1 year	0.81 y [0.79; 0.83]	0.86 y [0.84; 0.88]	0.86 y [0.85; 0.88]	0.81 y [0.79; 0.83]	$PB_{M}$ = 0.05 y [0.03, 0.08] ${\mathrm{PB}}_{M}^{\left(1\right)}$ = 0.003 y [−0.020; 0.027] $B_{{\hat{B}}_{M}<0}^{\mathrm{obs}}$ = −0.03 y [−0.08; 0.03]
RMST to 2 years	1.34 y [1.29; 1.38]	1.48 y [1.43; 1.52]	1.50 y [1.45; 1.54]	1.33 y [1.28; 1.37]	$PB_{M}$ = 0.17 y [0.11, 0.23] ${\mathrm{PB}}_{M}^{\left(1\right)}$ = 0.02 y [−0.04; 0.08] $B_{{\hat{B}}_{M}<0}^{\mathrm{obs}}$ = −0.12 y [−0.24; 0.01]

*Note:* $PB_{M}$ compares results after following the model versus going against the model. ${\mathrm{PB}}_{M}^{\left(1\right)}$ compares results after following the model with results after treating everyone. $B_{{\hat{B}}_{M}<0}^{\mathrm{obs}}$ shows the observed treatment effect in the subgroup of patients where the model predicted negative treatment effects.

### Out‐of‐Sample Performance of Model Fit in the Simulated Dataset

In Table A2 we see results for decision accuracy after using a 10‐fold CV procedure to obtain model estimates. By comparing with results in Table A1 we see overall worse performance, as expected.

**TABLE A2:** Measures for decision accuracy, for the simulated dataset (out‐of‐sample performance).

Estimand	Estimated value if treating…				Estimated population benefit
	No‐one	Everyone	With model	Against model	
Median survival	1.63 y [1.44; 1.76]	2.22 y [1.98; 2.53]	2.23 y [1.98; 2.53]	1.62 y [1.43; 1.75]	$PB_{M}$ = 0.61 y ${\mathrm{PB}}_{M}^{\left(1\right)}$ = 0.01 y $B_{{\hat{B}}_{M}<0}^{\mathrm{obs}}$ = −0.09 y
1‐year survival	64% [61%; 67%]	72% [69%; 75%]	72% [69%; 75%]	64% [62%; 67%]	$PB_{M}$ = 8% [4%; 12%] ${\mathrm{PB}}_{M}^{\left(1\right)}$ = 0% [−5%; 5%] $B_{{\hat{B}}_{M}<0}^{\mathrm{obs}}$ = −1% [−10%; 7%]
2‐year survival	43% [39%; 46%]	53% [49%; 56%]	53% [50%; 57%]	43% [40%; 46%]	$PB_{M}$ = 10% [5%; 15%] ${\mathrm{PB}}_{M}^{\left(1\right)}$ = 0% [−6%; 6%] $B_{{\hat{B}}_{M}<0}^{\mathrm{obs}}$ = −2% [−13%; 9%]
RMST to 1 year	0.81 y [0.79; 0.83]	0.86 y [0.84; 0.88]	0.81 y [0.79; 0.83]	0.86 y [0.84; 0.88]	$PB_{M}$ = 0.04 y [0.02, 0.07] ${\mathrm{PB}}_{M}^{\left(1\right)}$ = 0.00 y [−0.02; 0.02] $B_{{\hat{B}}_{M}<0}^{\mathrm{obs}}$ = −0.01 y [−0.06; 0.04]
RMST to 2 years	1.34 y [1.29; 1.38]	1.48 y [1.43; 1.52]	1.48 y [1.44; 1.52]	1.34 y [1.30; 1.39]	$PB_{M}$ = 0.14 y [0.08, 0.20] ${\mathrm{PB}}_{M}^{\left(1\right)}$ = 0.00 y [−0.06; 0.06] $B_{{\hat{B}}_{M}<0}^{\mathrm{obs}}$ = −0.03 y [−0.16; 0.10]

*Note:* $PB_{M}$ compares results after following the model versus going against the model. ${\mathrm{PB}}_{M}^{\left(1\right)}$ compares results after following the model with results after treating everyone. $B_{{\hat{B}}_{M}<0}^{\mathrm{obs}}$ shows the observed treatment effect in the subgroup of patients where the model predicted negative treatment effects.

### A Toy Example Illustrating Why Performance Measures Focused on Benefit Are Needed When Assessing Models for Benefit

In this section, we use a simulated example to show how it is not enough to assess the performance of predictions for outcomes in treatment and control to decide whether a model predicts benefit adequately. We show that a model might perform adequately for the former but may completely fail for the latter.

We simulate data similar to the ones described in the paper and in Appendix A.1, but with the rate of events slightly changed:

$$\begin{array}{cc} \mathrm{rat}e_{i} & =\exp\left(-1+0.3\;x_{1i}+0.\;3\;x_{2i}+0.2\;\left(x_{3i}==B\right)-0.2\;\left(x_{3i}==C\right)\right.\\ & \;\left.+0.1\;\left(x_{3i}==D\right)-0.1\left(x_{4i}==1\right)\right)\\ & \;+Z_{i}\left(-0.3+0.2\;x_{2i}+0.2\;(x_{3i}==B)\right) \end{array}$$

In essence, we have four prognostic variables, of which only two are effect modifiers ($x_{2},x_{3}$).

Let us now assume that we fit in the treatment and control group separately two identical models that only include $x_{1},x_{4}$. These two models include two important prognostic factors, but they completely miss the correct effect modifiers $x_{2},x_{3}$. After fitting the models, we use them to predict outcomes in‐sample, and assess their performance using measures that focus on outcome predictions. As expected, these models perform relatively well for predicting the absolute outcome in each of the treatment groups.

Discrimination according to Harrel's *C* statistic = 0.63, Uno's *C* statistic = 0.62.

Calibration slope 1.00, ratio of observed versus expected at time = 2 was estimated at 1.00.

Calibration plot:

Kaplan Meier curve per treatment group:

Based on these results, one might think that since the two models seem to perform well for both treatment groups, they would be adequate for predicting benefit. However, the two models do not use the effect modifiers at all, so they cannot predict benefit correctly. If we use the methods developed in this paper, we see that they completely fail to do so. For example:

Of course, these results in this example are not surprising, given the way that the data was generated and the models were developed. However, they serve to show that even if an algorithm performs well for predicting the outcome across both treatment groups, it does not necessarily follow that it also predicts well for predicting the treatment benefit.

R code used for the example in this section:

**# generate data ‐‐‐‐‐‐‐‐‐‐‐‐‐‐‐‐‐‐‐‐‐‐‐‐‐‐‐‐‐‐‐‐‐‐‐‐‐‐‐‐‐‐‐‐‐‐‐‐‐‐‐‐‐‐‐‐‐‐‐**
remove(list=ls())
library(survival)
set.seed(42) **# the answer to life the universe and everything**
options(digits=2)

**# simulate data ‐‐‐‐**
library(MASS)
N <‐ 2000
Sigma <‐ outer(1:4, 1:4, function(x,y) 0.5ˆabs(x‐y)) **#variance covariance matrix for covariates**

x <‐ mvrnorm(n = N, rep(0, 4), Sigma)
x[,3] <‐ cut(x[,3], breaks=c(‐Inf, ‐1, 1, 2, Inf)) **#categorical with 4 categories**
x[,4] <‐ ifelse(x[,4] > 0, 1, 0) #binary predictor

tr <‐ rbinom(size=1,n=N, p=0.5)
survdat <‐ data.frame(x,tr)
survdat[,3:5] <‐ lapply(survdat[,3:5], factor)
colnames(survdat)[1:4] <‐ paste0("x", 1:4)

survdat$rate0 <‐ with(survdat,
              exp(‐1+0.3*x1+0.3*x2+0.1*(x3=="2")
                 ‐0.2*(x3=="3")+0.1*(x3=="4")
                 ‐0.1*(x4=="1")))
survdat$benefit <‐ with(survdat,
               exp(‐0.3+0.2*x2+0.2*(x3=="1"))) # treatment benefit
survdat$rate1 <‐ with(survdat, rate0*benefit)

survdat$survivaltime0 <‐ rexp(N, rate = survdat$rate0)
survdat$survivaltime1 <‐ rexp(N, rate = survdat$rate1)

survdat$survival.with.treat.taken=with(survdat,
survivaltime0*(tr=="0")+survivaltime1*(tr=="1"))

censtimes <‐ 1 + 2*runif(N)
mean(censtimes)
survdat$time <‐ pmin(survdat$survival.with.treat.taken, censtimes)
survdat$status <‐ as.numeric(censtimes > survdat$survival.with.treat.taken)

**# fit models ‐‐‐‐‐‐‐‐‐‐‐‐‐‐‐‐‐‐‐‐‐‐‐‐‐‐‐‐‐‐‐‐‐‐‐‐‐‐‐‐‐‐‐‐‐‐‐‐‐‐‐‐‐‐‐‐‐‐‐‐‐‐**
f1 <‐ survreg(Surv(time, status)˜x1+x4,
         survdat[survdat$tr==1,], dist="weibul")
f0 <‐ survreg(Surv(time, status)˜x1+x4,
         survdat[survdat$tr==0,], dist="weibul")

**# assess predictive performance for outcome ‐‐‐‐‐‐‐‐‐‐‐‐‐‐‐‐‐‐‐‐‐‐‐‐‐‐‐‐‐‐‐**

lp0=c()
lp1=c()
for( i in 1:N){
  new.patient <‐ survdat[i,]
  lp0[i]<‐ predict(f0, newdata=new.patient, type="lp")
  lp1[i] <‐ predict(f1, newdata=new.patient, type="lp")
}

**# Assess apparent performance (no optimism correction) ‐‐‐‐‐‐‐‐‐‐‐‐‐‐‐‐‐‐‐**
calculate_performance <‐ function(time = NULL, status = NULL, lp = NULL){
  
  #discrimination
  harrell_C <‐ concordance(Surv(time, status) ˜ lp)
  harrell_C_est <‐ harrell_C$concordance
  #harrell_C_var <‐ harrell_C$var
  
  Uno_C <‐ concordance(Surv(time, status) ˜ lp, timewt = "n/G2")
  Uno_C_est <‐ Uno_C$concordance
  #Uno_C_var <‐ Uno_C$var
  
  **#calibration**
  second_model <‐ survreg(Surv(time, status) ˜ lp, dist="weibul")
  calslope <‐ second_model$coef[2]
  
  returnVec <‐ c(harrell_C_est, Uno_C_est, calslope)
  names(returnVec) <‐ c("Harrel_C", "Uno_C", "calibration.slope")
  
  return(returnVec)  
}  
apparent.weibull <‐ calculate_performance(time = survdat$time,
                         status = survdat$status,
                         lp = lp0*(survdat$tr==0)+lp1*(survdat$tr==1)
)
round(apparent.weibull,2)

**# calibration in the large**
t.0=2
**# Observed**
obj <‐ summary(survfit(
  Surv(time, status) ˜ 1,
  data = survdat),
  times = t.0)
obs_t <‐ 1 ‐ obj$surv

# **Predicted risk** 
prediction.weibull2 <‐ function(new.patient, fit.model = NULL, time.point = NULL){
  pct <‐ time.point
  predicted.exp <‐ c()
  mu_hat <‐ predict(fit.model, newdata = new.patient, type = "lp")
  predicted.exp <‐ 1 ‐ pweibull(pct, shape = 1/fit.model$scale,
                    scale = exp(mu_hat))
  
  predicted.exp.average <‐ mean( predicted.exp)
  
  results <‐ data.frame(time = pct, means = predicted.exp.average)
  return(results)
{

predictions0 <‐ list()
for (i in 1:dim(survdat)[1]){
  predictions0[i] <‐ prediction.weibull2(survdat[i,], f0, time.point = t.0)$means
}
predictions1 <‐ list()
for (i in 1:dim(survdat)[1]){
  predictions1[i] <‐ prediction.weibull2(survdat[i,], f1, time.point = t.0)$means
}

est.surv <‐ unlist(predictions0)*(survdat$tr==0)+unlist(predictions1)*(survdat$tr==1)
pred <‐ 1 ‐ est.surv

**# Expected**
exp_t <‐ mean(pred)
OE_t <‐ obs_t /exp_t

alpha <‐ .05
OE_summary <‐ c(
  "OE" = OE_t,
  "2.5 %" = OE_t * exp(‐qnorm(1 ‐ alpha /2) * sqrt(1 /obj$n.event)),
  "97.5 %" = OE_t * exp(+qnorm(1 ‐ alpha /2) * sqrt(1 /obj$n.event))
)
round(OE_summary,2)

**# calibration plot**
library(rms)
predictions0 <‐ c()
for (i in 1:dim(survdat)[1]){
  predictions0[i] <‐ prediction.weibull2(survdat[i,], f0, time.point = survdat[i,]$time)$means
}

predictions1 <‐ c()
for (i in 1:dim(survdat)[1]){
  predictions1[i] <‐ prediction.weibull2(survdat[i,], f1, time.point = survdat[i,]$time)$means
}

est.surv <‐ unlist(predictions0)*(survdat$tr==0)+unlist(predictions1)*(survdat$tr==1)
f <‐ val.surv(S= Surv(survdat$time, survdat$status),
              est.surv = est.surv)
plot(f, xlab="predicted survival", ylab="observed survival")

**# kaplan Meier curves per treatment group**
group0 <‐ survdat[survdat$tr == 0,]
group1 <‐ survdat[survdat$tr == 1,]

prediction.weibull <‐
  function(new.patient, fit.model = NULL){
    pct <‐ 1:200/100
    mu_hat <‐ predict(fit.model, newdata = new.patient, type = "lp")
    predicted.exp <‐ 1 ‐ pweibull(pct, shape = 1/fit.model$scale,
                      scale = exp(mu_hat))
    results <‐ data.frame(time = pct, means = predicted.exp)
    return(results)
  }  

predictions.group0 <‐ list()  **# predicted probabilities under control**
for (i in 1:dim(group0)[1]){
  predictions.group0[[i]] <‐
    prediction.weibull(group0[i,],f0)$means
}

predictions.group1 <‐ list() **# predicted probabilities under treatment**
for (i in 1:dim(group1)[1]){
  predictions.group1[[i]] <‐ prediction.weibull(group1[i,],f1)$means
}

predictions.mean.group1 <‐ apply(do.call(cbind, predictions.group1), 1, mean)
predictions.mean.group0 <‐ apply(do.call(cbind, predictions.group0), 1, mean)

predictions.both.groups <‐ data.frame(means=
c(predictions.mean.group0,predictions.mean.group1),
                       group= c(rep("Z=0, fitted", 200), rep("Z=1, fitted", 200)), percentiles.surv = 1:200/100)

m2 <‐ survfit(Surv(time, status) ˜ tr, data=survdat)
res <‐ summary(m2, censored = T)
dt1 <‐ with(res, data.frame(time = time, surv = surv, upper = upper,
                 lower = lower, strata=strata))

dt2 <‐ data.frame(time = c(1:200/100, 1:200/100, dt1$time),
                 surv = c(predictions.both.groups$means, dt1$surv),
                 group = c(predictions.both.groups$group, dt1$strata))
dt2$group[dt2$group == "1"] <‐ "Z=0, observed"
dt2$group[dt2$group == "2"] <‐ "Z=1, observed"

library(ggplot2)
ggplot(data = dt2) + 
  geom_line(aes(x = time, y = surv, group = group, colour=group, linetype=group,
           size = group)) +
  labs(x="Percent survival", y="Time (years)")+
  scale_size_manual(values=c(0.6,0.4,0.6,0.4))+
  scale_color_manual(values=c("black", "black","red", "red"))+
  scale_linetype_manual(values=c("solid", "dashed","solid", "dashed"))+
  xlim(0,2)+ theme(axis.text.y = element_text(size = 12))+ theme(axis.text.x = element_text(size = 12))+
  theme(axis.title = element_text(size = 14))

**# calibration for benefit ‐‐‐‐‐‐‐‐‐‐‐‐‐‐‐‐‐‐‐‐‐‐‐‐‐‐‐‐‐‐‐‐‐‐‐‐‐‐‐‐‐‐‐‐‐‐‐‐‐**
survdat$benefit.survival.at.fixed.time<‐ ‐100
for( i in 1:N)
{
  new.patient0 <‐ survdat[i,]; new.patient0$tr="0"
  new.patient1 <‐ survdat[i,]; new.patient1$tr="1"
  mu_hat0 <‐ predict(f0, newdata=new.patient0, type="link")
  mu_hat1 <‐ predict(f1, newdata=new.patient1, type="link")
  survdat$benefit.survival.at.fixed.time[i] <‐
    1 ‐ pweibull(t.0,shape=1/f0$scale,scale=exp(mu_hat1))‐(
      1 ‐ pweibull(t.0,shape=1/f1$scale,scale=exp(mu_hat0))) }

Ngroups=4
d1 <‐ quantile(survdat$benefit.survival.at.fixed.time, probs = seq(0, 1, 1/Ngroups))
g1 <‐ list()
for (i in 1:Ngroups) {
  g1[[i]] <‐ survdat[survdat$benefit.survival.at.fixed.time >= d1[i] & 
               survdat$benefit.survival.at.fixed.time < d1[i + 1], ]}

predicted <‐ c()
observed <‐ c()
SE.observed <‐ c()
lower.predicted<‐c()
upper.predicted<‐c()

library(survRM2 )
for (i in 1:Ngroups){
  km0 <‐ survfit(Surv(time, status)˜tr, data=g1[[i]][g1[[i]]$tr==0,])
  km1 <‐ survfit(Surv(time, status)˜tr, data=g1[[i]][g1[[i]]$tr==1,])
  observed <‐ c(observed,summary(km1, times=t.0)[6][[1]] ‐ summary(km0, times=t.0)[6][[1]])
  SE.observed <‐ c(SE.observed, sqrt(summary(km0, times=t.0)[7][[1]]ˆ2+summary(km1, times=t.0)[7][[1]]ˆ2))
  predicted <‐ c(predicted, mean(g1[[i]]$benefit.survival.at.fixed.time))
  lower.predicted <‐ c(lower.predicted,min(g1[[i]]$benefit.survival.at.fixed.time)  )
  upper.predicted <‐ c(upper.predicted,max(g1[[i]]$benefit.survival.at.fixed.time)  )
}

dat1 <‐ data.frame(pred = predicted, obs = observed,
                   SE.observed ,  lower.predicted , upper.predicted)

library(ggplot2)
suppressMessages(ggplot(dat1,aes(x=pred,y=obs))+
                geom_point(size=3,shape=20)+
                labs(x="Predicted benefit (survival probability at fixed time)", y="Observed benefit (survival probability at fixed time)")+
                geom_abline(intercept=0,slope=1,color="black",linetype="dashed",
                       size=0.5)+
                geom_errorbar(aes(ymin=obs‐1.96*SE.observed,ymax=obs+1.96*SE.observed,),width=0.01)+
                geom_errorbarh(aes(xmin=lower.predicted,xmax=upper.predicted), height=0.01)+
                geom_smooth(method="lm",
                       colour="blue",size=0.2)+theme(aspect.ratio=1))+xlim(c(‐0.05,0.25))+ylim(c(‐0.05,0.25))
